# From Nanothermometry to Bioimaging: Lanthanide-Activated KY_3_F_10_ Nanostructures as Biocompatible Multifunctional Tools for Nanomedicine

**DOI:** 10.1021/acsami.2c22000

**Published:** 2023-02-24

**Authors:** Chiara Cressoni, Federica Vurro, Emil Milan, Matilde Muccilli, Francesco Mazzer, Marco Gerosa, Federico Boschi, Antonello Enrico Spinelli, Denis Badocco, Paolo Pastore, Natalia Fernández Delgado, Miriam Herrera Collado, Pasquina Marzola, Adolfo Speghini

**Affiliations:** † Nanomaterials Research Group, Department of Biotechnology, University of Verona, Strada le Grazie 15, 37134 Verona, Italy; ‡ Division of Experimental Oncology, Urological Research Institute, IRCCS San Raffaele Scientific Institute, Via Olgettina 60, 20132 Milan, Italy; § University Vita-Salute San Raffaele, Via Olgettina 60, 20132 Milan, Italy; ∥ Department of Computer Science, University of Verona, Strada le Grazie 15, 37134 Verona, Italy; ⊥ Experimental Imaging Centre, San Raffaele Scientific Institute, Via Olgettina 60, 20132 Milan, Italy; # Department of Chemical Sciences, University of Padova, Via Marzolo 1, 35122 Padova, Italy; ∇ Department of Materials Science and Metallurgic Engineering and Inorganic Chemistry, University of Cadiz, Campus Universitario Río San Pedro, 11519 Puerto Real, Cádiz, Spain

**Keywords:** colloidal nanomaterials, lanthanides, optical thermometry, biocompatibility, bioimaging, multifunctional

## Abstract

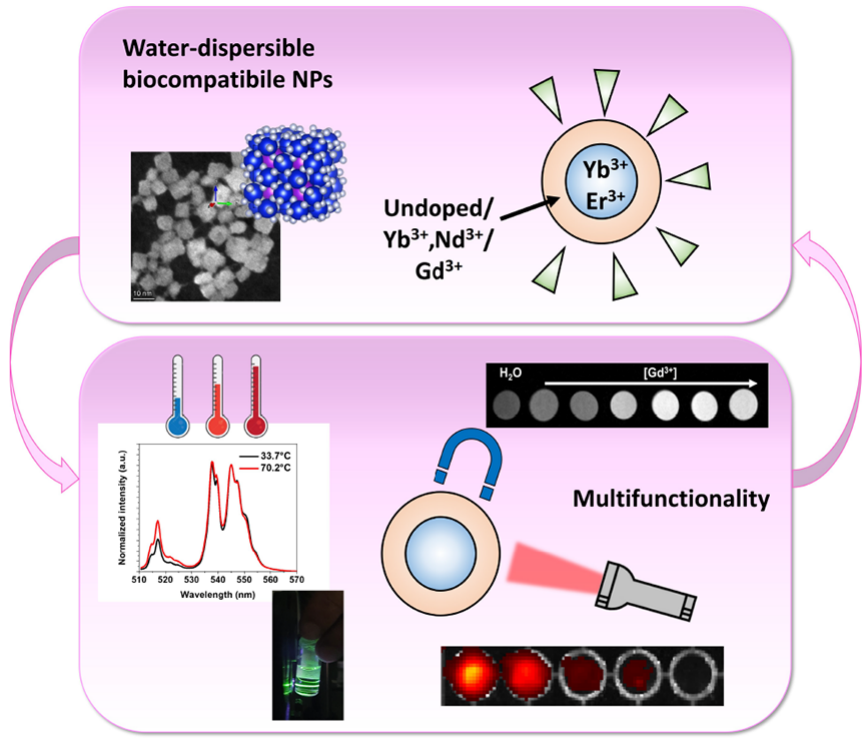

Lanthanide-activated fluoride-based nanostructures are extremely interesting multifunctional tools for many modern applications in nanomedicine, e.g., bioimaging, sensing, drug delivery, and photodynamic therapy. Importantly, environmental-friendly preparations using a green chemistry approach, as hydrothermal synthesis route, are nowadays highly desirable to obtain colloidal nanoparticles, directly dispersible in hydrophilic media, as physiological solution. The nanomaterials under investigation are new KY_3_F_10_-based citrate-capped core@shell nanostructures activated with several lanthanide ions, namely, Er^3+^, Yb^3+^, Nd^3+^, and Gd^3+^, prepared as colloidal water dispersions. A new facile microwave-assisted synthesis has been exploited for their preparation, with significant reduction of the reaction times and a fine control of the nanoparticle size. These core@shell multifunctional architectures have been investigated for use as biocompatible and efficient contrast agents for optical, magnetic resonance imaging (MRI) and computerized tomography (CT) techniques. These multifunctional nanostructures are also efficient noninvasive optical nanothermometers. In fact, the lanthanide emission intensities have shown a relevant relative variation as a function of the temperature, in the visible and near-infrared optical ranges, efficiently exploiting ratiometric intensity methods for optical thermometry. Importantly, in contrast with other fluoride hosts, chemical dissolution of KY_3_F_10_ citrate-capped nanocrystals in aqueous environment is very limited, of paramount importance for applications in biological fluids. Furthermore, due to the strong paramagnetic properties of lanthanides (e.g., Gd^3+^), and X-ray absorption of both yttrium and lanthanides, the nanostructures under investigation are extremely useful for MRI and CT imaging. Biocompatibility studies of the nanomaterials have revealed very low cytotoxicity in dfferent human cell lines. All these features point to a successful use of these fluoride-based core@shell nanoarchitectures for simultaneous diagnostics and temperature sensing, ensuring an excellent biocompatibility.

## Introduction

The research interest in biocompatible hosts with multifunctional properties is growing nowadays, due to the urgent need of new materials for applications in nanomedicine. Bioimaging techniques are widely used in biomedical practice, both in clinical and medical research contexts, but every technique suffers from several disadvantages, each one related to the type of imaging approach,^1^ as reported in Table 1.

**Table 1:** Comparison between the Most Common Imaging Techniques Used for Theranostics and Some Examples of Nanomaterials Used As Contrast Agents (CA)

bioimaging technique	probe	source	advantages	disadvantages	NPs ^[ref]^
optical/fluorescence imaging	photon emission	laser/lamp	high sensitivity	low penetration depth	UCNPs^2^
			multichannel acquisition	low spatial resolution	QDs^3^
magnetic resonance imaging	proton relaxation	magnetic field	high spatial resolution	high costs	IONPs^4^
			unlimited penetration depth	high acquisition time	Gd-MSNs^5^
				relative low sensitivity	
computed tomography	scattered X-rays	X-rays source	unlimited penetration depth	non quantitative	Micelles^6^
			high spatial resolution	exposure to radiation	
positron emission tomography	positron	X-rays source	high sensitivity	high cost	PEG-based NPs^7^
			quantitative	exposure to radioactive elements	
			unlimited penetration depth		

The most promising and potential evolution in bioimaging deals with the development of nanosized materials, which can combine different functionalities.^2^ Nanomaterials can be prepared by engineering their structure to obtain increased sensitivity, specificity, as well as multimodal response for several bioimaging techniques. A low toxicity of the prepared nanoparticles (NPs) remains a milestone for their use as *in vivo* contrast agents. Moreover, attention should be paid to preparation methods, involving green chemistry methods, with low reaction temperature and reduced reaction time.^2^

Lanthanide (Ln^3+^)-doped nanomaterials are particularly interesting for their typical luminescence properties in the UV, visible, and near-infrared (700–1200 nm) regions, due to their peculiar energy level structure, making them ideal materials for optical bioimaging and temperature sensing.^3,8^ In addition, due to their unpaired electrons in the outer configuration 4f shell, they have relevant paramagnetic properties, making Ln^3+^-doped nanoparticles optimal also as MRI contrast agents.^9^ Moreover, Ln^3+^ ions have notable X-ray absorption cross sections in the typical regions employed in modern computed tomography (CT), suggesting their use as X-ray contrast agents.^10^ Recently, Ln^3+^ activated nanomaterials have raised interest in nanotheranostics due to their excellent performance in deep-seated tumors,^11^ and they have been also demonstrated to be extremely useful for the generation of reactive oxygen species (ROS) for photodynamic therapy (PDT).^12^ The peculiar ladder-like energy level structure of the Ln^3+^ ions is suitable for the generation of radiation at higher energies with respect to the excitation radiation, through the upconversion (UC) process, recently investigated for promising applications in biomedicine.^13^

Fluoride-based hosts are suitable for accommodating luminescent Ln^3+^ ions, as their low-phonon energies are ideal to enhance the luminescence with respect to lanthanide organic complexes,^9^ due to reduced nonradiative multiphonon relaxations.

Among the extensively investigated lanthanides-activated upconverting nanomaterials, the most famous is certainly the ternary host NaYF_4_, which crystallizes in two possible phases, namely, cubic and hexagonal one.^14^ Nonetheless, the KY_3_F_10_ ternary host has been scarcely investigated, although it has excellent optical properties. In fact, the luminescence of Tm^3+^-, Nd^3+^-, Yb^3+^-, and Er^3+^-doped KY_3_F_10_ nanopowders were investigated by Gomes et al.,^15^ highlighting excellent Ln^3+^ emissions upon infrared excitation for the Tm^3+^-, Nd^3+^-, Yb^3+^- or Er^3+^-codoped samples. Most notably, in these studies, high values of luminescence efficiency were found.^16^ Colloidal dispersions of oleate-capped and Ln^3+^-doped KY_3_F_10_ NPs, prepared by a thermolysis procedure, were also investigated by Mahalingham et al.^17^ It is worth noting that the Ln^3+^ dopant ions are accommodated in low symmetry sites (*C*_4v_) of the KY_3_F_10_ crystal structure, with relatively high transition probabilities.^18,19^ Recently, the UC thermometric performance of Er^3+^, Yb^3+^-doped core@shell KY_3_F_10_ nanopowders (particle size of 60–70 nm) were investigated, by Solanki et al.,^20^ and a high thermal sensitivity value has been found. Besides, Er^3+^- and Yb^3+^-codoped KY_3_F_10_ NPs have been prepared by an hydrothermal synthesis using ethylenediaminetetraacetic acid (EDTA)^21^ (particle size around 38 nm), for which a strong emission at 1530 nm upon laser excitation at 980 nm has been evidenced.

Another important aspect to consider is that biocompatibility of inorganic nanoparticles, developed for biomedical purposes, is strongly affected by their chemical and colloidal stability, as well as their possible chemical dissolution, thus determining the dispersion of potentially toxic ions within the organism.^22^ For this reason the chemical stability of water dispersible NPs should be systematically investigated, in combination with the cell viability assays, for a more comprehensive study before possible *in vivo* applications. This aspect is particularly important also considering the limited amount of available stability data for inorganic nanostructures.^23,24^

**Figure 1:**
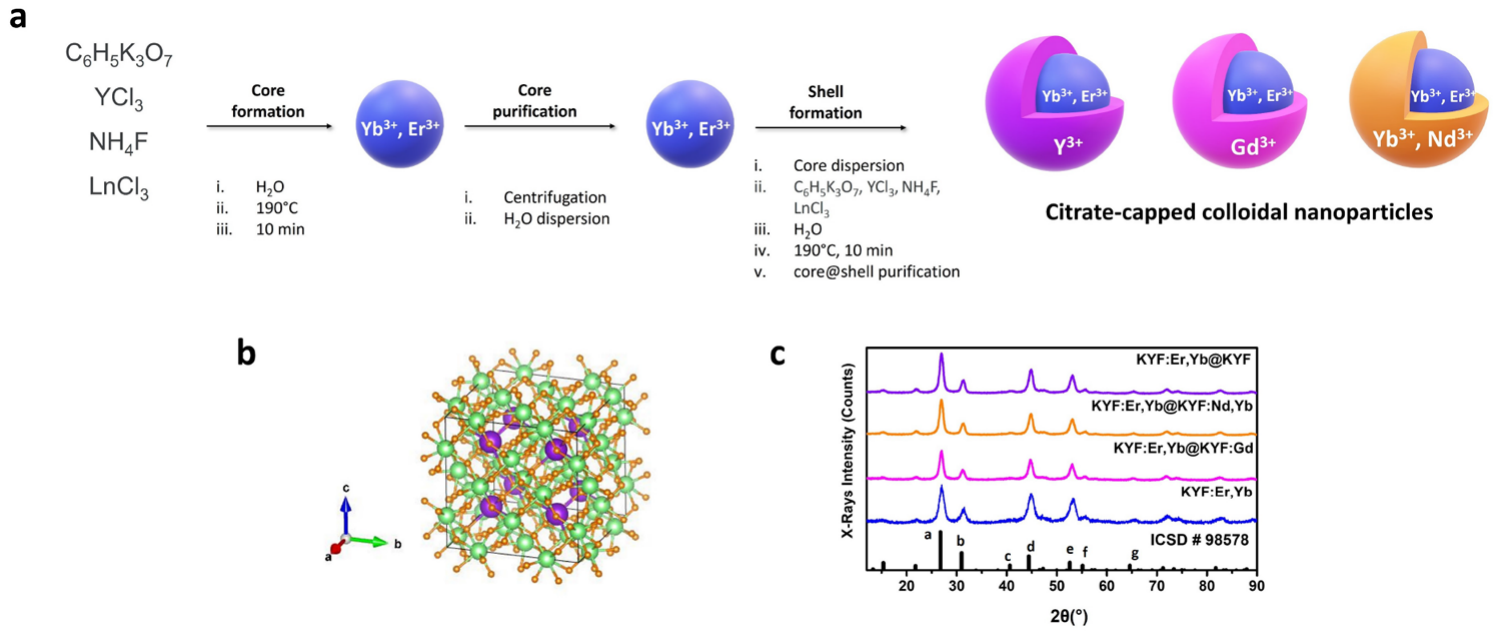
(a) Schematic representation of the facile preparation and the core@shell architecture of the KY_3_F_10_ NPs. (b) Sketched crystal structure of undoped KY_3_F_10_ matrix (K in violet, Y in green, F in orange). (c) X-rays powder diffraction patterns for core and core@shell nanoparticles, ICSD reference: a (222); b (400); c (151); d (404); e (226); f (444); g (080).

To the best of our knowledge, no detailed studies have been published about colloidal multifunctional nanostructures based on the ternary KY_3_F_10_ host. Therefore, in the present investigation, we focused on this host, doped with Ln^3+^ ions, in stable colloidal form, for applications in nanomedicine, prepared with a microwave-assisted *green chemistry* procedure. In order to maximize the synergy among the dopant ions, core@shell architectures were considered, depicted in Figure 1a.

Briefly, the main objective of this paper is to develop a comprehensive study about this interesting versatile biocompatible material, to put solid bases for feasible applications in nanomedicine, especially by exploiting commercially available imaging facilities commonly used in diagnostics.

## Results and Discussion

### Structural and Morphological Aspects

The produced nanoparticles (see Section S0 in the Supporting Information for synthetic details) are readily dispersible in aqueous environment, confirming the presence of hydrophilic citrate moieties as capping agent on the particle surface, and the particle sizes and the morphologies have been investigated through different techniques. The chosen architecture for the core@shell nanoparticles is represented in Figure 1a and, from now on, the KY_3_F_10_ NPs will be denoted using the following conventional names: the Er^3+^,Yb^3+^-doped core KY_3_F_10_ NPs are denoted in the paper as KYF:Er,Yb; the KY_3_F_10_:Er^3+^,Yb^3+^@KY_3_F_10_ NPs are denoted as KYF:Er,Yb@KYF; the KY_3_F_10_:Er^3+^,Yb^3+^@KY_3_F_10_:Gd^3+^ NPs are identified as KYF:Er,Yb@KYF:Gd; and the KY_3_F_10_:Er^3+^,Yb^3+^@KY_3_F_10_:Nd^3+^,Yb^3+^ NPs are denoted in the paper as KYF:Er,Yb@KYF:Nd,Yb.

**Figure 2:**
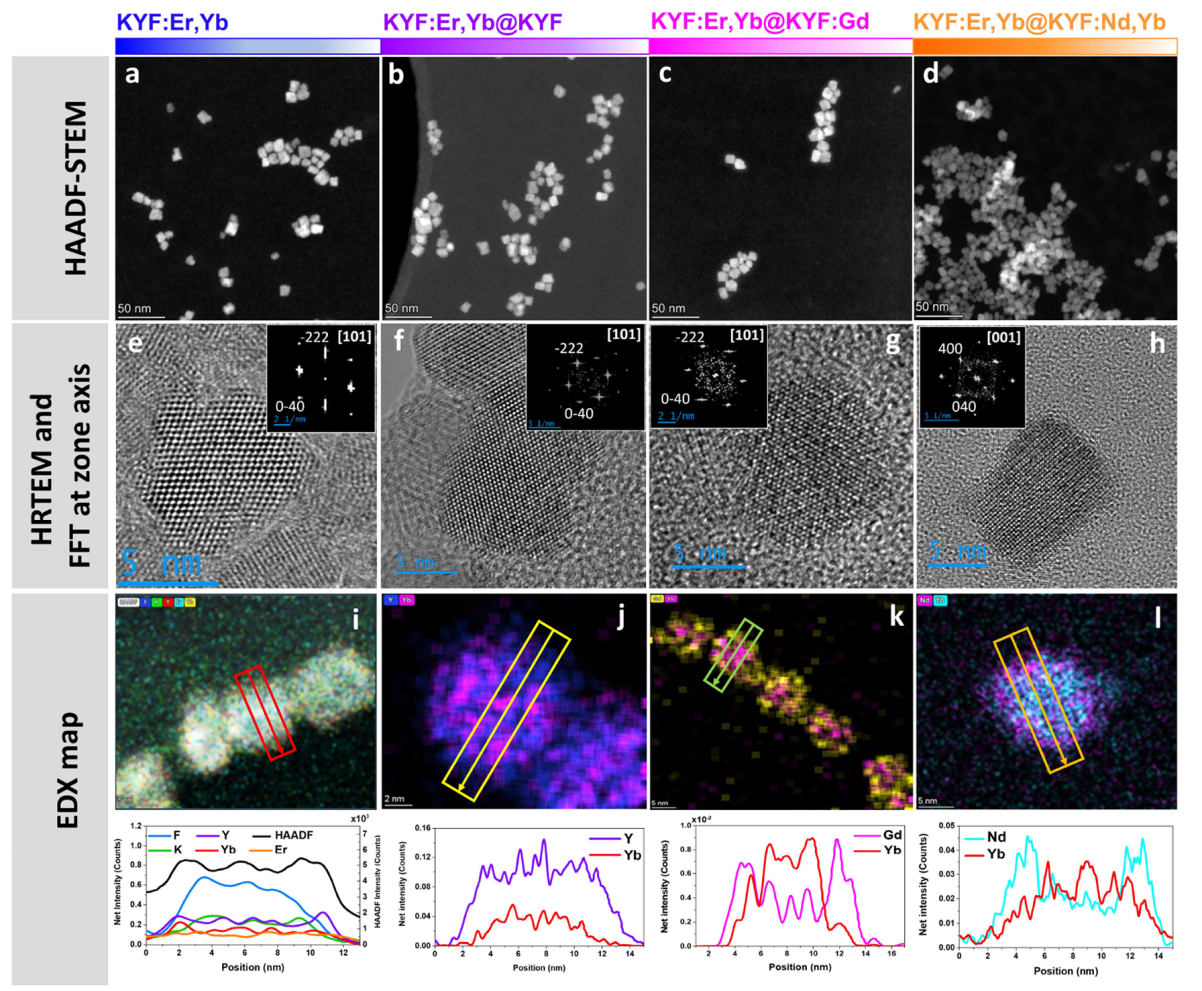
(a–d) EM analysis of the core and core@shell nanoparticles: HAADF-STEM images. (e–h) High resolution TEM micrographs and relative FFT patterns at zone axis. (i–l) EDX maps and relative EDX intensity profiles along the rectangles.

Transmission electron microscopy (TEM) images of the prepared Ln^3+^-doped KY_3_F_10_ NPs, demonstrate the nanosized nature of both core and core@shell architectures, as it can be noted in Figure 2 and in Figure S1 in the Supporting Information. Thus, measurements of the size distribution carried out in high angle annular dark field-scanning (HAADF-S) TEM images (averaging 100 nanoparticles for each sample) show that core nanoparticles have sizes of approximately 6 nm, whereas core@shell architectures exhibit larger average diameters of 10–11 nm (see Section S1 of the Supporting Information). From the HAADF-STEM images at low magnification (Figure 2a–d), it is possible to appreciate the morphology of the nanocrystals, appearing nearly cubic in shape and homogeneous. High resolution (HR-)TEM and the corresponding FFT analysis (Figure 2e–h) demonstrate that the nanoparticles are monocrystalline.

Regarding the dopants, it is worth noting that HAADF-STEM is an excellent tool for the analysis at atomic column level of the composition distribution in the material, as the intensity in this technique is related to the average atomic number (Z) of the material. Because of this relation with Z, it is expected that atomic columns where rare-earth dopants are present show higher intensity than others without dopants (or with smaller amount).

High resolution HAADF-STEM imaging has been carried out on the KYF:Er,Yb@KYF sample, in order to analyze the presence of an undoped shell.

The results obtained for this core@shell suggest the presence of a shell with less average Z around of some nanoparticles, as it can be observed in Figure S2 of the Supporting Information, where regions with less HAADF-STEM intensity are visible around the nanoparticles (marked with yellow stars). Actually, these low contrast regions are not observed in other samples. The presence of an undoped shell is corroborated by EDX analysis as reported in Figure 2j.

High resolution HAADF-STEM imaging has also been carried out in KYF:Er,Yb@KYF:Nd,Yb nanoparticles. As it can be clearly appreciated in Figure S3, adjacent atomic columns show significant intensity variations, which can be related to the presence of rare-earth dopants. In order to obtain further information regarding the dopants distribution, EDX maps have been obtained for all elements (Figure 2i) or by choosing one element from the core and one element from the shell (Figure 2j–l). In order to clarify the visualization of these results, intensity EDX profiles have been taken along a single nanoparticle, and the resulting curves clearly follow the expected distribution of dopants within the nanoparticles.

Actually, the average particle sizes of the observed nanostructures obtained derived from TEM images (Section S1 in the Supporting Information) are in very good agreement with the values from dynamic light scattering (DLS) measurements, which have been carried out on colloidal dispersions (Figure S5 and Table T1, Supporting Information).

Additionally, the measured hydrodynamic diameters and ζ-potentials of the NPs in colloidal dispersions remain constant for several days, clearly demonstrating their stability. Typical ζ-potentials for such nanocrystals are lower than −30 mV (see Figure S5 and Table T1 in the Supporting Information), confirming the colloidal stability of the colloids.

The measured XRD patterns for the core and core@shell nanoparticles are shown in Figure 1c and Section S3 of the Supporting Information, together with the theoretical undoped one, calculated from cubic KY_3_F_10_ (Inorganic Crystal Structure Database, ICSD, n. 98578, space group *Fm*3̅*m*) and represented graphically in the Figure 1b. A comparison of these XRD patterns clearly shows that all the nanostructures are single phase. An analysis of the experimental XRD patterns using the Bragg law (eqs S3 and S4) for a cubic structure is reported for the core KYF:Er,Yb NPs (Section S3 of the Supporting Information, Figure S6 and Table T2). It is worth noting that the calculated lattice constant parameter (*a* = 11.4184 ± 0.0054 Å) results to be much lower than that for KY_3_F_10_ single crystal (*a* = 11.54398 Å at 295 K^25^). This behavior can be explained with the presence of a large amount of Yb^3+^ and Er^3+^ doping ions, which substitute the Y^3+^ ions within the crystal lattice. The ionic radii of these dopants (114.4 pm for Er^3+^ and 112.5 pm for Yb^3+^, in 8-fold coordination^26^), are smaller than for Y^3+^ (115.9 pm, 8-fold coordination^26^), and therefore, the heavily doped sample presents a smaller lattice unit cell. The XRD patterns for the core@shell NPs, shown in Figure 1c, also match very well the ICSD pattern for cubic KY_3_F_10_, demonstrating that they also are single cubic phase and that the shell is constituted by the same host matrix KY_3_F_10_ epitaxially grown on the surface of the core NPs.

### Luminescence Features of Colloidal Dispersions

**Figure 3:**
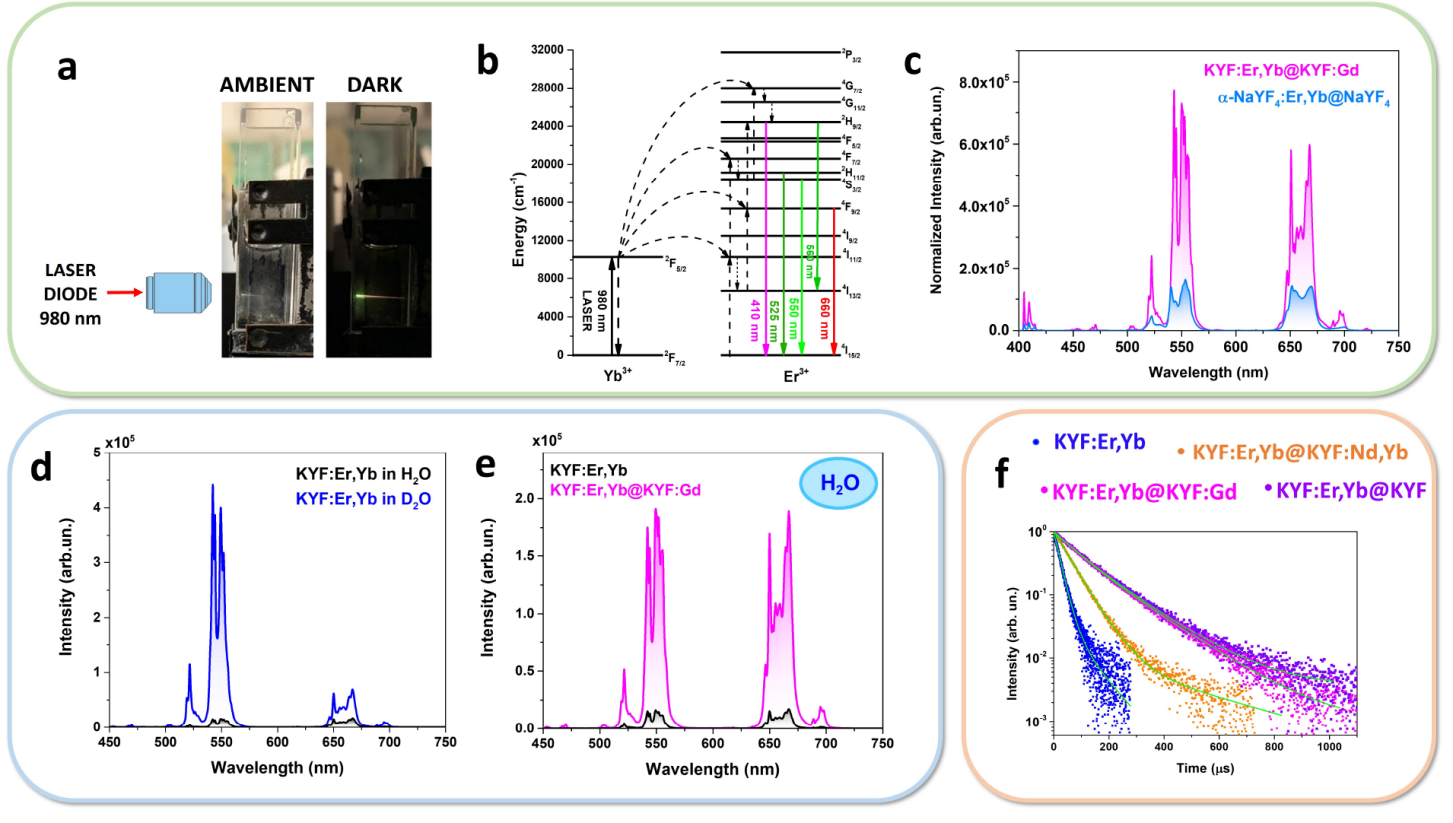
(a) Snapshots of the UC emission for a colloidal dispersion of KYF:Er,Yb@KYF:Gd (concentration of 20 mg/mL). (b) Energy level scheme for Yb^3+^ and Er^3+^ ions and UC mechanisms. (c) UC emission spectra of KYF:Er,Yb@KYF:Gd and α-NaYF_4_:Er,Yb@NaYF_4_ in colloidal dispersion, normalized on Yb^3+^ concentration, under 980 nm laser excitation, *P* ∼ 4 kW cm^–2^. (d) UC spectra of colloidal dispersions of core KYF:Er,Yb (20 mg/mL) in H_2_O and D_2_O. (e) UC spectra (λ_exc_ = 980 nm) for colloidal dispersions of core KYF:Er,Yb (20 mg/mL) and core@shell KYF:Er,Yb@KYF:Gd (20 mg/mL) in H_2_O. (f) Emission decays in the green due to the ^2^H_11/2_, ^4^S_3/2_ →^4^I_15/2_ transitions of Er^3+^ (λ_exc_ = 980 nm, λ_em_ = 543 nm) for all the nanostructures (concentration of 20 mg/mL).

The upconversion (UC) emissions in the visible–NIR range for all the Er/Yb-codoped KYF nanostructures have been investigated in colloidal dispersions (concentration of 20 mg/mL) under 980 nm NIR laser excitation. The typical UC emission visible at naked eye is shown in Figure 3a for the illustrative KYF:Er,Yb@KYF:Gd sample, where a yellowish-green UC light is clearly observed.

A comparison among the UC spectra of the core and core@shell nanostructures is reported in Figure S8 (Supporting Information). The emission spectra evidence the typical emission bands of to Er^3+^ ions in the green–yellow region, from 520 to 575 nm. The group of emission bands in the 520–550 nm range is assigned to the ^2^H_11/2_, ^4^S_3/2_ →^4^I_15/2_ transitions, involving the ^2^H_11/2_, ^4^S_3/2_ as emitting levels (see Figure 3b and Figure S8 in the Supporting Information). Besides, it is worth remarking that another excited level is involved in the generation of emission bands in the 550–575 nm range. In fact, as recently highlighted by Xia et al.,^27^ bands in the latter range are also due to radiative transitions starting from the ^2^H_9/2_ excited level, precisely assigned to the ^2^H_9/2_ →^4^I_13/2_ transition (see Figure 3b and Figure S8 in the Supporting Information). Moreover, in the red region (640–700 nm), bands due to ^4^F_9/2_ →^4^I_15/2_ transitions are observed. It is therefore evident that the Yb^3+^ ions act as sensitizers, absorbing exciting radiation at 980 nm and efficiently populating the ^2^F_5/2_ excited level of Yb^3+^ ions *via* the electronic transition ^2^F_7/2_ →^2^F_5/2_. Then, energy transfer processes take place from Yb^3+^ to Er^3+^ ions through the ^4^I_11/2_ excited levels of Er^3+^ (see Figure 3b and Figure S8). The UC mechanism involves two consecutive energy transfers (two-photon processes), populating the green and red emitting states of Er^3+^ ions.^17^

Moreover, it is worth to highlight that a high emission brightness for the nanomaterials is required in order to be considered as luminescent contrast agents in nanomedicine.

Therefore, we found it interesting to carry out a comparison between the UC luminescence of the as-prepared colloidal dispersion of KY_3_F_10_ core@shell nanostructures and an illustrative sample of the well-known NaYF_4_ host,^28^ also in core@shell architecture. In order to be consistent with the *green chemistry* synthetic approach followed in this study, a one-step microwave-assisted hydrothermal technique, using water as a solvent has been also considered to prepare NaYF_4_ for the comparison. Nonetheless, the preparation of NaYF_4_-based nanoparticles with a size comparable to the studied KYF nanostructures using an hydrothermal procedure, produces NaYF_4_ nanoparticles only in α-phase, while β-NaYF_4_ NPs can be prepared with the same procedure but with a much larger size.^29^

Thus, α-NaYF_4_ nanoparticles doped with 20% Yb^3+^ and 2% Er^3+^ have been prepared through the same microwave-assisted synthetic procedure and a doped-core/inert-shell architecture has been chosen for a fair comparison (see Section S0 and Figure S7 for the preparation details and XRD patterns, respectively, in the Supporting Information). From a DLS analysis, an size around 20 nm (see Figure S4) has been estimated for the core@shell α-NaYF_4_ NPs. As shown in Figure 3c, the Gd-doped KYF core@shell NPs present more than a 3-fold increase of the luminescence intensity with respect to that observed for the α-NaYF_4_ core@shell NPs, normalized by the absorption of Yb^3+^ ions. Moreover, a 2-fold increase for the Nd-doped core@shell NPs has been found, while a comparable emission intensity is observed for the KYF NPs with undoped shell (see Figure S8c in the Supporting Information).

Furthermore, we also consider it useful to study the influence of the solvent, namely, water, on the upconversion luminescence intensities and decays of our colloidal UC nanophosphors. Evidently, due to the very small size of the core KYF:Er,Yb nanoparticles (around 6 nm), the percentage of lanthanide ions on the nanoparticle surface is very high with respect to the total amount in the whole nanoparticle. For aqueous colloidal dispersions, the luminophors are located directly in contact with the water molecules, which are very efficient luminescence quenchers *via* multiphonon relaxation processes. In order to investigate the emission quenching, UC spectra of core KYF:Er,Yb NPs dispersed in H_2_O and in D_2_O have been measured, taking advantage of the very different vibrational energies of these two solvents. In fact, H_2_O presents higher energy vibrations (vibrational energy cutoff around 3600 cm^–1^) than D_2_O (vibrational energy cutoff around 2600 cm^–1^).^30^ The comparison between UC in the two solvents is shown in Figure 3d, evidencing that the UC intensity is much higher for the KYF:Er,Yb NPs colloids in D_2_O than in H_2_O. Actually, the ratio between the integrated UC intensities in D_2_O and in H_2_O in the visible range is more than an order of magnitude (increasing by a factor of 11.7). This behavior demonstrates a strong luminescence quenching due to solvent molecules close to the lanthanide emitters situated on the nanoparticle surface. Moreover, from a detailed analysis of the UC spectra, an interesting difference between the behavior for the core KYF:Er,Yb NPs in the two solvents is evidenced. In fact, the relative intensities of the UC bands in the red and green regions are different. Precisely, the red-to-green ratio of the UC for the KYF:Er,Yb NPs is 1.2 in H_2_O (black line in Figure 3d), very different from the value of 0.21 found for the same nanoparticles in D_2_O dispersion (blue line in Figure 3d). The high increase in the red-to-green UC ratio in the transition from D_2_O to H_2_O can be explained by different population dynamics of the ^2^H_11/2_,^4^S_3/2_, and ^4^I_9/2_ emitting levels. In fact, the OH– groups facilitate the multiphonon relaxation of the ^2^H_11/2_, ^4^S_3/2_ levels to the ^4^I_9/2_ ones as well as that of the ^4^I_11/2_ level to the ^4^I_13/2_ one, in a more remarkable way with respect to the OD– groups. Both these OH-dependent relaxations increase the population of the ^4^F_9/2_ level, responsible for the red emission, and therefore, the red-to-green ratio results much lower in D_2_O than for H_2_O.^31^

In order to further investigate the improvement of the luminescence efficiency of the core@shell nanostructures with respect to the core only, the UC spectra of the KYF:Er,Yb (core) and KYF:Er,Yb@KYF:Gd (core@shell) aqueous dispersions have been recorded (Figure 3e). Due to the core@shell architecture, for a given mass percentage of the dissolved whole nanostructures, the luminophore concentrations could be different for core and core@shell dispersions. In order to directly compare the relative emission efficiencies for the two samples, a normalization factor with respect to the molar concentration of the emitting lanthanide ions is evaluated by comparing the absorption bands around 980 nm of the Yb^3+^ ions for the different nanostructures (see Figure S9 in the Supporting Information). The normalization factors are calculated by integrating the absorption bands and dividing each UC spectra by these values.

From the normalized spectra, it can be noted at a glance that the shell structure has a very important role on increasing the emission efficiency. As a matter of fact, the integrated ratio between the UC intensities for the core@shell and core NPs results in more than an order of magnitude (precisely, an increasing factor of 11.6), clearly demonstrating a very high improvement of the emission efficiency in aqueous dispersions after the shell growth. Therefore, the lanthanide luminescence quenching due to multiphonon de-excitation mediated by water vibrations is much less pronounced than for the simple core NPs.^32^

It has to be observed that the red-to-green ratio of the UC for the KYF:Er,Yb@KYF:Gd (core@shell) in H_2_O (fuchsia line in Figure 3e) is 1.2, in perfect agreement with the value found for the KYF:Er,Yb (core) in H_2_O.

It is worth noting that the shell thickness for the KYF:Er,Yb@KYF:Gd NPs has been estimated, from the TEM analysis of around 2.5–3 nm. This luminescence improvement behavior is similar but proportionally more pronounced than that found for much bigger nanoparticles, around 56 nm (core) and 70 nm (core@shell), made of the same Er,Yb-doped host.^20^ For such NPs, a 2-fold increase in the overall fluorescence intensity of the core@shell NPs with respect to the core-only ones is observed. Although a direct comparison is difficult due to the very different sizes of the nanostructures, a possible explanation for the different behavior could be in the distinct experimental conditions, as in our case the UC is measured in colloidal dispersions instead of on powder sample, as in the case of Solanki et al.^20^ It seems reasonable that, in a water environment, the luminescence quenching is very effective and mainly due to solvent molecules in direct contact with the active lanthanide ions on the surface of the NPs. For the sake of completeness, in Figure S10 (left part), a comparison of the UC spectra between KYF:Er,Yb (core) NPs (blue line) and KYF:Er,Yb@KYF:Gd (core@shell) ones (red line) dispersed in D_2_O is shown. In this case, the ratio between the integrated UC emission for core@shell and core samples is 5.6, lower than that found for the same couple of samples in H_2_O. This behavior is reasonable and it is due to the lower phonon energy cutoff of D_2_O with respect to H_2_O. Moreover, in Figure S10 (right part), the UC of the KYF:Er,Yb@KYF:Gd NPs is shown for the two different solvents, and a value of 5.9 has been found for the ratio between the integrated UC emissions for core@shell and core samples.

From this behavior, it can be concluded that, although the shell growth around the core nanoparticle is strongly beneficial to intensify the emission of the nanoparticles by more than order of magnitude in H_2_O, the fact that we observe almost a 6-fold increase of the UC on passing from H_2_O to D_2_O suggests that the shell is not perfectly shielding all the lanthanide ions on the particle surface, probably due to some ion migrations during the thermal treatment for the shell growth. Nonetheless, a possible further improvement of the luminescence efficiency for the present NPs would be growing an additional or a thicker and possibly more homogeneous shell around the core.

In order to further investigate the improvement of the luminescence of the nanostructures after the growth of the shell, decay curves of the lanthanide emission have been measured and a lifetime analysis is carried out. In particular, decay curves for both the green and red UC emissions of Er^3+^ ions upon pulsed 980 nm laser excitation have been acquired. The decay curves for the green and red emissions for the core and core@shell nanostructures, corresponding to the ^4^S_3/2_ →^4^I_15/2_ transition (λ_em_ = 543 nm) and ^4^F_9/2_ →^4^I_15/2_ transition (λ_em_ = 650 nm), respectively, are shown in Figure 3f and Figure S11, respectively. The decay curves are well-fitted using a biexponential function (eq S1 in the Supporting Information), and the calculated decay times of the two components together with the corresponding weight factors and the average lifetimes (eq S2) are reported in Tables T3 and T4 in the Supporting Information. The obtained average lifetime for the green emission for core sample is 31.6 μs. This value is much lower than the one found for nanopowders of KY_3_F_10_:Yb(20%),Er(0.5%) (73.5 μs) reported by Gomes et al.,^15^ although in this case the Er^3+^ concentration in the nanoparticles is much lower, with reduced cross-relaxation processes that favor a lengthening of the lifetime; moreover, in our case, the small nanoparticle size and water molecules around the nanoparticles lead to a faster multiphonon decay rate with respect to free-standing nanoparticles in air. Reasonably, the lifetime for the core sample is also much lower than the one measured for a Er^3+^ (0.97%) single-doped KY_3_F_10_ single crystal (261 μs), reported by Labbé et al.^33^ It is clear from the Figure 3f and Table T3, that a remarkable 4-fold increase of the average decay time for the ^4^S_3/2_ Er^3+^ level is observed, on passing from the core to the core@shell structure, with exception for the KYF:Er,Yb@KYF:Nd,Yb, for which a 2-fold increase was observed, due to some back transfer processes occurring between Er^3+^ and Nd^3+^, confirmed by the presence of emission from Nd^3+^ ions under 980 nm excitation, shown in Figure S8 (right picture). In the same way, the decay times for the red emission (see Figure S11 and Table T4) show a significant increase after shell growing on the core nanoparticles, following similar decay mechanisms than for the green emission. Therefore, it is worth underlying the very high improvement of the luminescence efficiency in colloidal dispersions of the KYF:Er,Yb@KYF:Gd NPs with respect to the core ones, that, together with their very stable colloidal properties, corroborate the possibility of useful applications in nanomedicine as efficient luminescent contrast agents.

In order to fully understand the behavior of the UC luminescence of the investigated nanoparticles, the power density (P) dependence of red and the green upconversion bands has been evaluated, under the assumption that the intensity of upconversion luminescence is a consequence of the absorption of *n* photons, and as a general rule, the absorbed pump power is defined as P_*n*_. Two excitation power ranges have been considered, in order to consider an exhaustive range of possibilities, and UC spectra and P dependence plots are reported in Figures S12 and S13, respectively, together with the complete discussion (Section S6 in the Supporting Information). In addition, it is worth noting that the Nd^3+^ ions are able to absorb radiation around 800 nm. Therefore, in the case of the KYF:Er,Yb@KYF:Nd,Yb nanostructures, the Nd^3+^ can act as sensitizer, opening the possibility to obtain upconversion under 800 nm laser excitation, by exploiting the energy transfer from Nd^3+^ to Yb^3+^ and then to Er^3+^ (see energy diagram and energy transfer mechanisms in Section S7, Supporting Information), leading to UC Er^3+^ emission in the visible range by excitation within the first biological window of transparency (Figure S14).

### Nanothermometry of Colloidal Dispersions

The thermometric properties of prepared UCNPs water dispersion have been investigated in different portions of the electromagnetic spectrum, precisely exploiting the UC emission from Er^3+^ in the visible range and the down-shifting emission of Nd^3+^ and Yb^3+^ in the near infrared region, in a wavelength range from 850 to 1000 nm. In order to develop efficient optical thermometers for biological applications, it is of paramount importance to take into consideration the possibility of working with excitation and/or emission radiation within the biological windows, which would reflect in several advantages in terms of tissue penetration depth and biological damage.^34^

#### NIR-to-Vis UC Thermometry and Induced Heat

From the upconversion spectra under 980 nm laser excitation, thermometric measurements for the KYF:Er,Yb@KYF nanostructures have been performed by exploitation of thermosensitive radiative transitions ^2^H_11/2_ →^4^I_15/2_ and ^4^S_3/2_ →^4^I_15/2_ of the Er^3+^ ion, centered at 517 and 540 nm respectively, within the visible region. The thermometric parameter, thermal relative sensitivity, and the temperature uncertainty have been calculated as reported in Section S8 on the Supporting Information. It is known that the potential use of optical nanothermometers as diagnostic tools in biological systems is limited by the thermal properties of tissues and by the tissue ability to dissipate laser-induced heating.^35^ For this reason, we have examined the case of 980 nm laser irradiation, used for performing UC optical thermometry. On the other hand, absorption of 980 nm radiation induces some heating by means of vibrational relaxation. Thus, we found it useful to estimate the temperature variation of the sample, due to heating induced by the laser beam during the thermometric measurement. This aspect is important to assess the reliability of the optical thermometer, especially for *in vivo* applications. For this reason, a calculation of the temperature variation by the exciting laser on water dispersion has been performed (see Section S9 in the Supporting Information). The estimated temperature variation for our experimental setup and conditions is 0.04 °C. Therefore, we can consider this temperature variation negligible for our experimental conditions, which generate an excellent signal-to-noise ratio of the UC spectra (see Figure S15a). As a further proof of concept of the reliability of our thermometric analysis, an experiment using a biological tissue, namely, a chicken breast slice, has been carried out, for estimation of the temperature variation in a biological tissue under laser excitation at 980 nm (see Section S9). After 50 s of CW 980 laser radiation, the maximum induced temperature variation is close to 1 °C (Figures S17 and S18). Then, this temperature variation is similar to the minimum temperature uncertainty evaluated for the KYF:Er,Yb@KYF core@shell nanothermometer (Δ*T*_min_ = 1 °C, see Section S8), indicating that the heating induced by the laser at 980 nm is negligible for real experimental conditions in biological tissues.

#### Nd^3+^-Yb^3+^ NIR Thermometry

**Figure 4:**
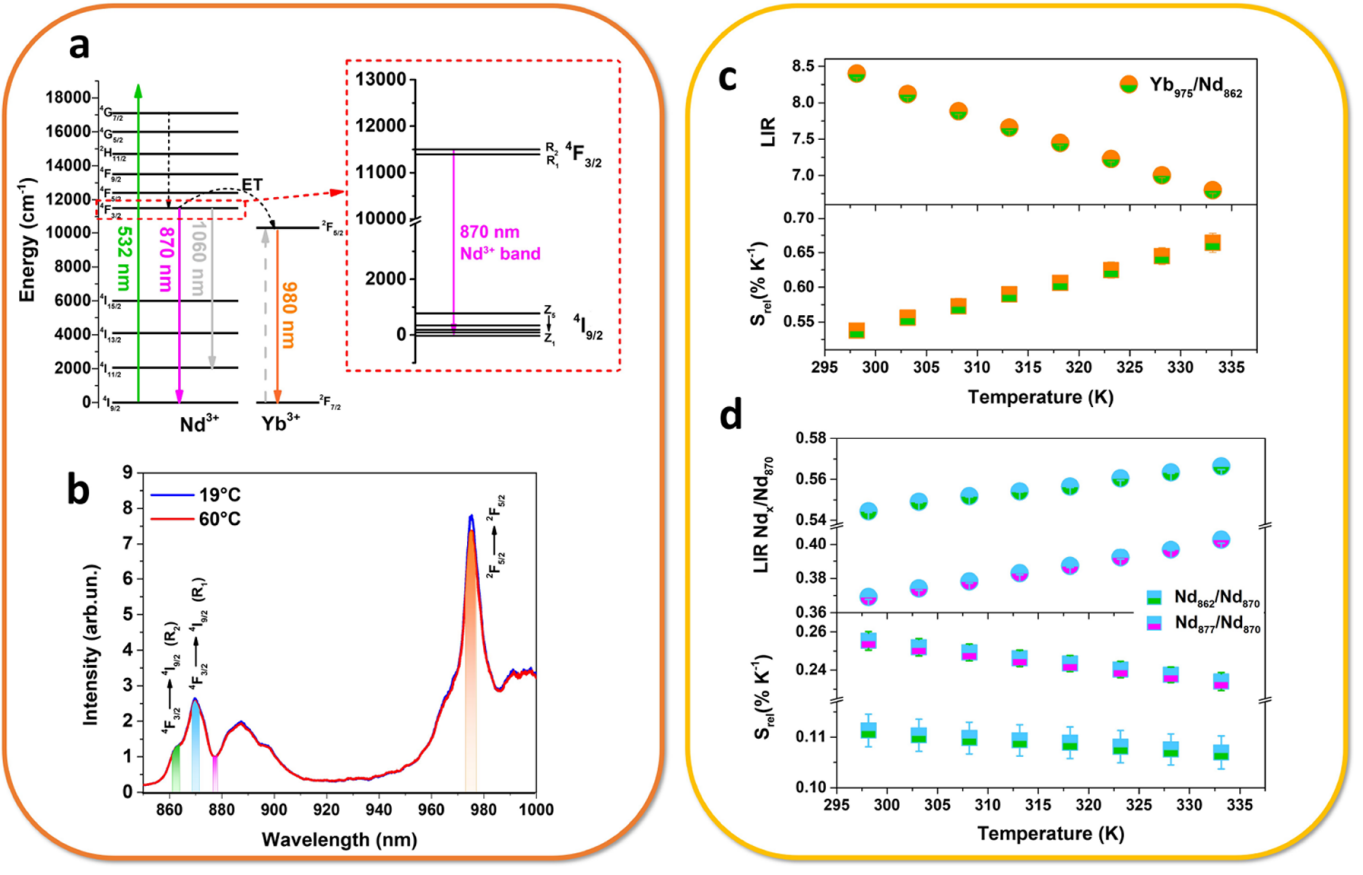
Thermometric features: (a) energy level scheme for the Nd^3+^ and Yb^3+^ ions, with radiative and energy transfer processes, (b) emission spectra as a function of temperature and the LIR variation, and the relative sensitivity for the (c) Yb/Nd transitions and (d) Nd/Nd levels.

The KYF:Er,Yb@KYF:Nd,Yb nanoparticles have been investigated as vis-to-NIR optical thermometers under laser excitation in the visible range at 532 nm (see Figure 4a). Although not in the optical biological windows, this wavelength has been chosen for investigation of the thermometric performances as a proof of principle, because this radiation is capable of exciting the Nd^3+^ ions with a very large Stokes shift of the emission band around 850 nm, therefore permitting a very good spectral resolution. The thermometric properties of such colloidal nanoparticles in water dispersion have been investigated by measuring the luminescence spectra as a function of the temperature, within the biological range of interest, 20–60 °C (Figure 4b).

Figure 4c clearly shows that the intensity ratio between the emission of Yb^3+^ and Nd^3+^ decreases with the increasing in temperature, since the thermometric properties rely on the fact that the Yb^3+^ emission strongly decreases with respect to a small increasing in the Nd^3+^ emission. For this reason, the luminescence intensity ratio (LIR) in this case is defined as
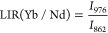
1where *I*_λ_ corresponds to the integrated areas, shown in orange and green in Figure 4c, for the emissions centered at 976 and 862 nm, belonging to ^2^F_5/2_ →^2^F_7/2_ and ^4^F_3/2_ →^4^I_9/2_ transitions of Yb and Nd, respectively.

Precisely, this behavior has been previously described by Cortelletti et al.,^36^ as they have shown that, similarly to other previously reported works,^37^ the thermometric properties of these system depend on the presence of a phonon assisted mechanism of nonresonant energy transfer (ET), according to the Miyakawa–Dexter (MD) model.^38^ Moreover, as previously demonstrated, the temperature behavior of the Yb^3+^ ion also depends on the presence of Er^3+^ ions: by increasing the temperature, the population of the energy Stark levels of the Er^3+^ ground state (^4^I_15/2_) increases, leading to an increased energy transfer between the Yb^3+^ and Er^3+^ ions. Consequently, the population of the Yb^3+2^F_5/2_ level decreases, producing a decrease in the emission at 980 nm. As a consequence, it is reasonable that the decreasing trend of Yb/Nd LIR is also dependent by the depopulation of the lowest ^2^F_5/2_ Stark level of Yb^3+^, and this effect should increase with the increase of the Er^3+^ concentration.

The *S*_rel_ is therefore calculated for LIR(Yb/Nd) (Figure 4c, bottom part). For allowing a quantitative comparison of the thermometric performance in a biological environment, the relative thermal sensitivity is here reported at the physiological body temperature (310 K), in order to settle on the biological application of these nanoparticles.^39^ Following eq S7 in the Supporting Information, the calculated value of the maximum *S*_rel_ is 0.57% K^–1^ at 310 K. This value can be compared with the values previously found in Cortelletti et al.,^36^ where they report values between 0.4 and 1.6% K^–1^ at different Er^3+^ concentrations for a SrF_2_ colloidal host. Finally, it is worth noting that usually the Yb/Nd ratio is calculated by using emission at 980 nm for Yb^3+^ and at around 1060 for Nd^3+^ for thermometric measurements. These bands generally lead to a better performance, but their recording could also be limited by the commercially available detector acquisition range, so the investigation of thermometry using other emission bands could be extremely useful for wider applications.

Besides, we also consider for thermometric purposes a LIR between Nd^3+^ energy levels only (see Figure 4d). In particular, we take into consideration the emission bands due to the ^4^F_3/2_ →^4^I_11/2_ Stark transitions (see the inset in Figure 4a). More in detail, we evaluate the ratio between emission bands due to the R_1_ → Z_1_ and R_2_ → Z_1_ transitions and additionally the ratio between the R_1_ → Z_1_ transition and the minimum of the ^4^F_3/2_ →^4^I_11/2_ band, which can be resumed in the following equations:
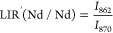
2
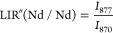
3

The R_1_ and R_2_ energy levels of Nd^3+^ are thermometrically coupled^40^ and they offer the advantage to be easily distinguished in the emission band (see Figure 4b), facilitating the thermometric analysis. Practically, the thermometric relative sensitivity increases when the difference between the integrated bands at two different temperature increases, meaning that the slope increases. This can be easily verified by calculation of relative sensitivity (eq S7 in Section S8). The *S*_rel_ is therefore calculated for such ananosystem for both LIR′ and LIR″ (see Figure 4d, bottom part).

Futhermore, the *S*_rel_ values for LIR(Nd/Nd) (Figure 4d) are found equal to 0.11% and 0.25% K^–1^ at 310 K for Nd_862_/Nd_870_ (LIR′) and Nd_877_/Nd_870_ (LIR″), respectively.

Additionally, from the distance between bands in the spectrum, the Δ*E* between the R_1_ and R_2_ Stark levels of Nd^3+^ can be calculated, and it is found equal to 93 cm^–1^. Neglecting the energy transfer processes occurring between the adjacent lanthanide ions and thus assuming, as in the case of Er^3+^ energy levels (Figure S16 in the Supporting Information), that the emission intensities of the two Nd^3+^ transitions are only proportional to the population of the corresponding energy states, the Boltzmann distribution law can be used to define the intensity ratio, as shown also in eq S6 in the Supporting Information.^41^ Under this assumption and through performing straightforward mathematical calculations, the theoretical relative sensitivity for Nd_862_/Nd_870_ band ratio is established to be equal to 0.16% K^–1^. This value is very similar to those found empirically, and it is also in good agreement with other previously reported Nd-based colloidal nanothermometers, mostly showing *S*_rel_ values between 0.1 and 0.25% K^–1^.^40,42^

Moreover, other two meaningful parameters can be calculated from the thermometric data in order to better evaluate the performance but also the reliability of the nanothermometer in aqueous environment: the temperature uncertainty, Δ*T*_min_, and the repeatability, *R*.^43^ Δ*T*_min_ represents the uncertainty in the measurement of the temperature, so the smallest temperature resolvable by the thermometer. Since it strongly depends on the type of sample and on the measurement setup, it is calculated here by using the error in the LIR calculation, coming from the standard deviation in the LIR determination at each temperature, measured a few times. Such an error is called ΔLIR and can be used to calculate Δ*T*_min_ by means of the equation:
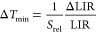
4

The average obtained value of Δ*T*_min_ for the NIR nanothermometers, representing the smallest temperature uncertainty that can reasonably be discriminated from the thermometric measurements, is reported in Table T5 (Section S10) in the Supporting Information. On the other hand, the repeatability (*R*) is an evaluation of the reliability of the measure in repeated heating–cooling cycles. This parameter can be calculated from the formula:
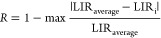
5where at the numerator there is the distance between the experimental values of LIR and the average LIR, taken at its maximum value (maximum distance between these two points). As a representative data, the value of *R* found for the LIR′(Nd/Nd) thermometry is around 98%, assessing that the nanothermometer is able to provide a very accurate result under the same measurements conditions (see Table T5).

### Chemical and Colloidal Stability Monitored by UC Emission

As previously mentioned, an important issue that has to be carefully considered when choosing a crystalline host for use in nanomedicine is its chemical stability in aqueous dispersion. Very recently, the dissolution behavior in aqueous media of NaYF_4_, one of the most investigated fluoride hosts for the incorporation of luminescent Ln^3+^ ions, has been investigated by several authors.^23,24,44−46^ These studies highlight that differently capped NaYF_4_ NPs can partially or even totally dissolve in aqueous media, depending on the capping moieties and on their concentration. This behavior points to severe limitation on the possible use of NaYF_4_ NPs in biological fluids. For these reasons, we found it interesting to investigate the chemical stability of the as-prepared citrate-capped KY_3_F_10_ colloids in both water and cell culture media, by monitoring the UC emission intensity as a function of their concentration and time. In fact, upconversion emission intensity strongly depends on the stability of the colloidal dispersion, since a decrease in time of the luminescence, keeping constant all the other experimental conditions, could be related to the dissolution of the nanocrystals.^44^

**Figure 5:**
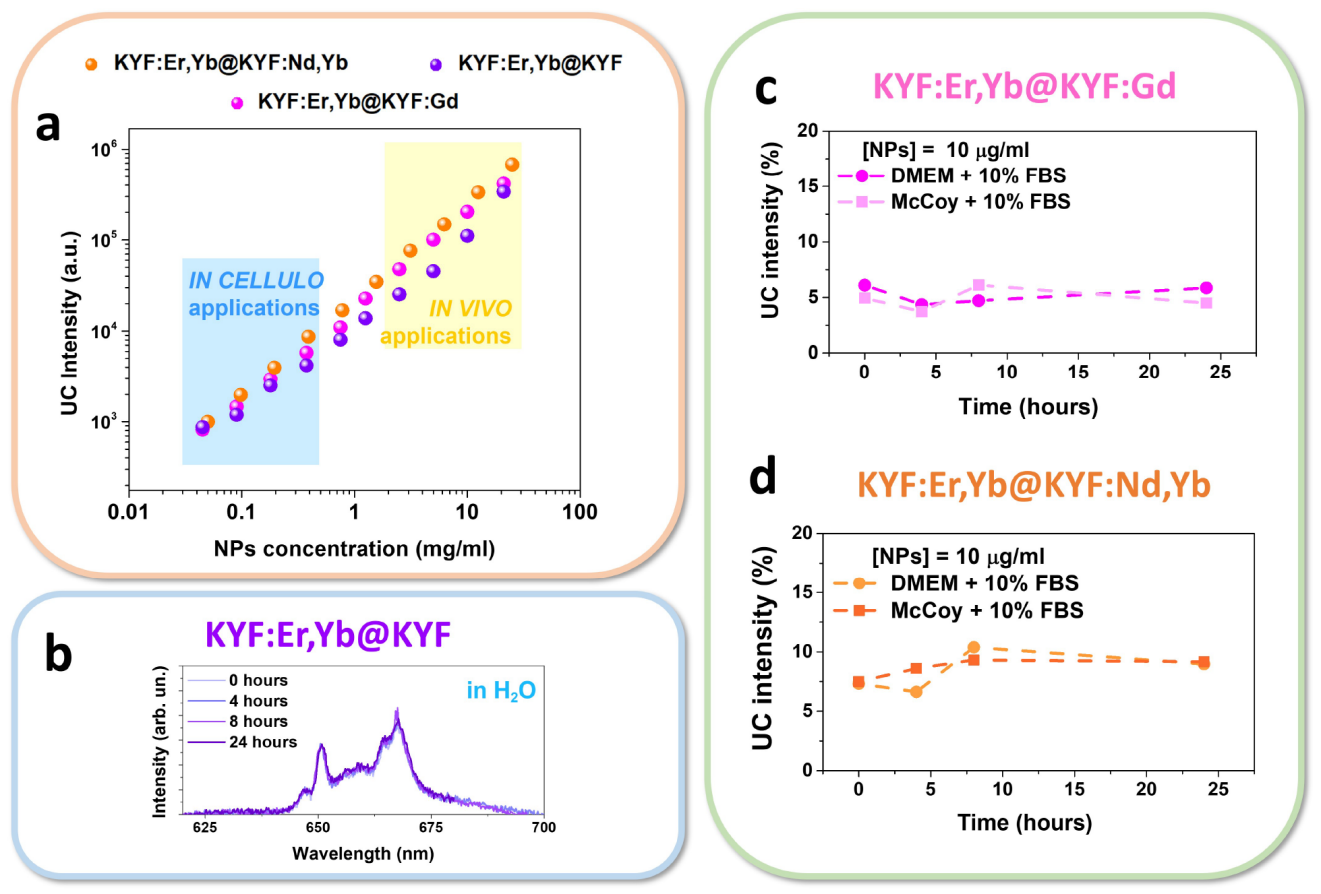
Intensity of upconversion emissions of core@shell nanoparticles under 980 nm laser excitation as a function of (a) NPs concentration and (b) time, in water dispersions. (c and d) Integrated upconversion emission in Dulbecco’s minimum essential medium (DMEM) and McCoy’s medium, both modified with 10% of fetal bovine serum (FBS).

Thus, the stability of the colloidal dispersions of the present KYF core@shell nanoparticles was first evaluated following the variation of UC luminescence intensity, by measuring the emission spectra of serial dilution of NPs aqueous dispersion and keeping constant the experimental setup and acquisition parameters for each measurement (Figure 5a).

The citrate-capped UCNPs were kept at room temperature and the dispersions were diluted with deionized water up to a minimum concentration of around 50 μg/mL, which is the concentration at which NaYF_4_ nanoparticles dissolve in water.^44^ All the core@shell NPs present linear correlation between UC emission and concentration, also at very low nanoparticles concentrations. As shown in Figure 5a, two ranges of concentrations can be identified and correlated with the well-assessed *in vitro* and *in vivo* range of nontoxic concentrations.^47^ Also, it has been previously reported that the chemical stability of water-dispersible citrate-capped NaYF_4_ UCNPs is very low at concentrations below 100 μg/mL, with luminescence that decreases of more than 90% after 5 h.^48^ Therefore, the stability of KYF core@shell nanoparticles at the lowest detectable concentration in water dispersion has been tested, by diluting the initial dispersion up to 10 μg/mL, corresponding to the minimum concentration at which the upconversion emission can be still distinguished (see red emission band around 660 nm in Figure 5b). UC luminescence has been acquired toward time, from the dispersion preparation up to 24 h later. Virtually, no variation of UC luminescence is found after 24 h in acqueous dispersion, as shown in Figure 5b, demonstrating a very low solubility for the KY_3_F_10_ crystalline structure and at the same time a high chemical stability of the surface capping moieties. To deeply investigate the stability of such nanosystems in media of biological interest, the UC luminescence from core@shell nanoparticles was further tested in widely used cell culture media DMEM and McCoy, at the minimum concentration of 10 μg/mL. As reported in Figure 5c,d, the UC intensity is almost constant after 24 h at room temperature in both cell culture media. It is worth remarking the importance of this behavior, clearly pointing to a successful application in aqueous environments, as well as in biological fluids, of paramount importance for applications in nanomedicine or in biological studies.^44^

### Cell Viability and Confocal Imaging

**Figure 6:**
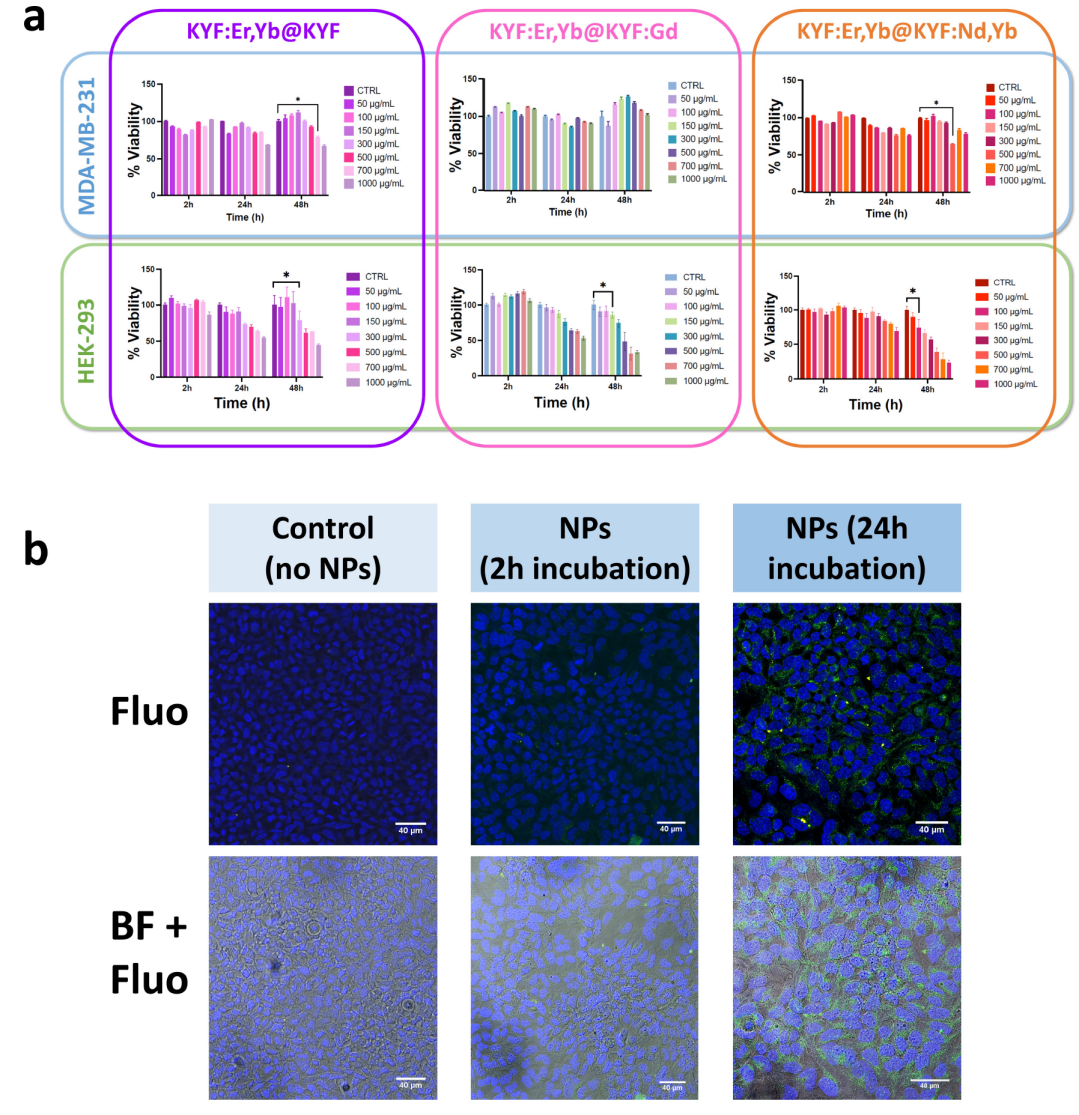
(a) MTT assay results for two human cell lines (one healthy and one tumoral). Cell cultures have been incubated with different concentrations of NPs and vitality tests have been performed after 2, 24, and 48 h. For each graph, the * indicates the first significative concentration, which resulted to be toxic to the cell line. (b) Confocal images of control (nontreated) HEK cells and HEK cells incubated with KYF:Er,Yb@KYF:Gd NPs after 2 and 24 h of treatment, under diode laser excitation at 405 nm for Hoechst (blue region 410–460 nm) and a 514 nm laser diode for NPs (green 520–590 nm).

Assessing the toxicity toward human cell lines for the translation of NPs into clinical research and medical applications is of great importance, due to the very high potential of such nanomaterials in bioimaging and nanomedicine.^49^ Significant variability of cytotoxic effect of nanomaterials is reported in the literature, depending on the nature of NPs, their concentration, and also the cell type.^50^ The main goal of the present cytotoxicity study is to determine the maximum nontoxic concentration of NPs that could be used for biomedical applications. As a general consideration, evaluating a specific cellular target is not the main topic of this work; thus, biocompatibility toward both healthy and cancer cell lines, differently distributed among the body, has been tested, by considering cellular models commonly used in biomedical research. For this purpose, MTT assay has been selected to evaluate the possible cytotoxic effect of different concentrations of KYF:Er,Yb@KYF, KYF:Er,Yb@KYF:Gd, and KYF:Er,Yb@KYF:Nd,Yb colloidal nanoparticles on MDA-MB-231 and HEK-293 human cell lines, as reported in Figure 6a.

MDA-MB-231 is a highly aggressive, invasive, and poorly differentiated triple-negative breast cancer (TNBC) cell line, as it lacks estrogen receptor (ER) and progesterone receptor (PR) expression, as well as HER2 (human epidermal growth factor receptor 2) amplification.^51^ On the other hand, human embryonic kidney (HEK-293) cells have been chosen as a healthy cell model, previously used in the literature for toxicity studies on novel molecular or nanostructured compounds.^52,53^

Cell viability has been assessed after 2, 24, and 48 h of incubation with the three as-prepared core@shell NPs dispersions at different concentrations (ranging from 50 to 1000 μg mL^–1^). MTT assays reveal the maximum nontoxic concentration for each selected cell line. It has been found that, for the MDA-MB-231 cell line, KYF:Er,Yb@KYF is safe up to 500 μg mL^–1^, KYF:Er,Yb@KYF:Gd is safe up to the concentration of 1000 μg mL^–1^, while KYF:Er,Yb@KYF:Nd,Yb is safe up to the concentration of 300 μg mL^–1^ also for long incubation times (48 h), as shown in Figure 6a. On the contrary, HEK cells demonstrate to be more sensitive toward NPs dispersion and the concentrations, which resulted to be nontoxic are 150 μg mL^–1^ for KYF:Er,Yb@KYF, 100 μg mL^–1^ for KYF:Er,Yb@KYF:Gd, and 50 μg mL^–1^ for KYF:Er,Yb@KYF:Nd,Yb. The differences in toxic concentrations of NPs are clearly dependent by the cell type: MDA-MB-231 cancer cells exhibit a higher resistance to the exogenous substances and thus present higher tolerance to NPs concentrations. On the other hand, embryonic healthy HEK-293 cells are known to be very sensitive cell line so they show a higher cytotoxicity also at lower NPs concentrations. It is worth noting that for both cell lines a maximum nontoxic concentration has been found for further use. Additionally, as a proof-of-concept of the possibility of exploiting functional NPs also in other cells, three other commonly used human cell cultures have been tested for one intermediate concentration of NPs, for assessing their nontoxic effect at the given concentration (see Figure S19 in the Supporting Information). In particular, HT-29, a human colon cancer cell line, which is extensively used for cancer research,^54^ has been used. Moreover, PANC-1 is a human pancreatic cancer cell line isolated from a pancreatic carcinoma of ductal cell origin and human lung fibroblast cell line, namely, HLF-1 cells were chosen as an additional healthy cell line.

It is important to underline that for all the evaluated core@shell nanoparticles a safe concentration has been identified, without the need of further surface modification, not only for cancer cells but also on healthy cell lines, which are usually much more sensitive that the tumoral ones. These results are not only extremely interesting for acquiring knowledge on nanotoxicology but also for putting solid bases for future use of these NPs in bioimaging or diagnostic applications, also for the very challenging cancer research. Moreover, a confocal microscopy imaging experiment was performed by exploiting healthy human HEK cells, treated with KYF:Er,Yb@KYF:Gd core@shell NPs, for a more extensive investigation of the possible internalization capability. They were chosen because an interesting dose-dependent cytotoxicity after 24 and 48 h of incubation was observed in the MTT assay, as reported in Figure 6a. From confocal images of the HEK cells, shown in Figure 6b, internalization of nanoparticles has been visualized. By using the maximum nontoxic concentration at the selected time-points (150 μg/mL for 24 h incubation, corresponding to the first time-point after a complete cell cycle), internalization of Gd-doped colloidal nanocrystals after 24 h of incubation has been verified. In fact, the green emission is mainly localized around the nucleus and in correspondence of cytoplasm. The study of the internalization of the NPs within the cells is particularly interesting for understanding the NPs faith and their toxicity, as well as the possibility of being used as aspecific cell markers for imaging and biomedical applications.^55^

### Performance of the Core@Shell Nanostructures As Multimodal (Optical, MRI, and CT) Imaging Contrast Agents (CA)

The lack of significant toxicity in cellular models of the three different of core@shell nanoparticles has encouraged us to deeply investigate the possible applications of such materials in nanomedicine, especially as bioimaging tools.

#### Quantitative Investigation Using Colloidal Acqueous Dispersions

**Figure 7:**
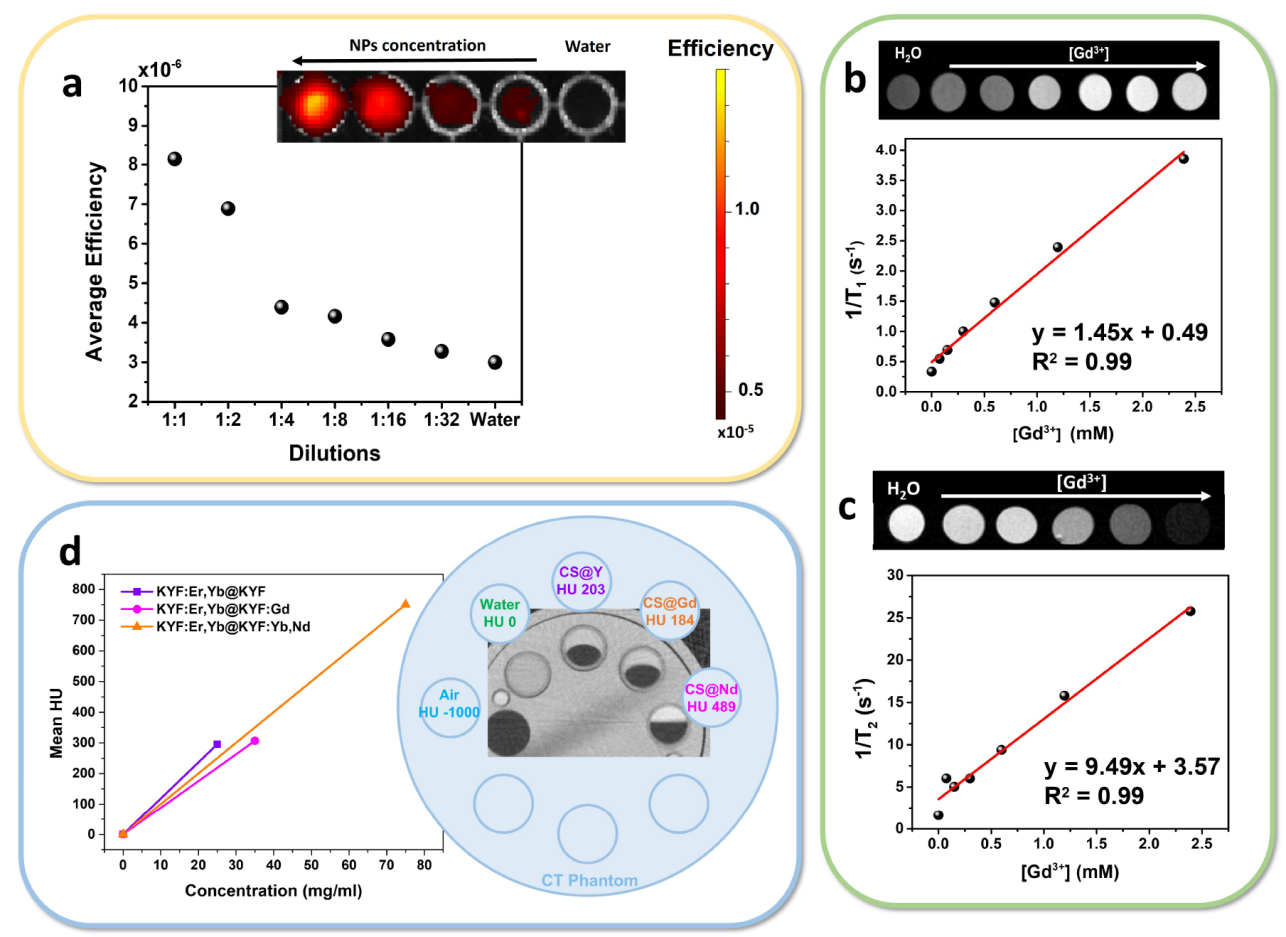
(a) Optical imaging efficiency of the KYF:Er,Yb@KYF:Nd,Yb NPs dispersions in the NIR range (1:1 dilution corresponds to 50 mg mL^–1^). (b and c) Evaluation of core@shell Gd^3+^-doped KY_3_F_10_ performance for MRI. Inverse of the longitudinal *T*_1_ and transverse *T*_2_ relaxation times vs the Gd^3+^ concentration. Linear fit for relaxivity determination: red line. Slopes of the fit: (b) *r*_1_ and (c) *r*_2_ relaxation rate constants. (d) Image: X-ray attenuation of KYF core@shell nanoparticles dispersions in a suitable phantom. Graph: interpolation of the X-ray attenuation vs nanoparticles concentration at 40 kVp (the line is a guide for the eye).

Thus, in order to demonstrate the possibility for these core@shell nanoparticles of being efficient multifunctional imaging CAs, as well as potential platforms for the development of diagnostic tools, combinations of imaging techniques were used, by exploiting optical and paramagnetic properties of the nanomaterials, and their performances were investigated (Figure 7).

Optical imaging using photons emitted within the first biological transparency window was performed by exploiting the Stokes emission of core@shell nanoparticles doped with Nd^3+^. The sample has been illuminated with a radiation at 570 nm, exciting the lanthanide ions from the ground state to the ^4^G_5/2_, ^2^G_7/2_ energy levels (see Figure 4a). The produced Stokes emission in the first biological window, from 850 to 1000 nm, was employed for the optical imaging. In fact, it is worth noting that the Nd^3+^ ions act not only as emitters but also as sensitizers, transferring the absorbed energy to the Yb^3+^ ions, acting in this case as activators (as a consequence of energy transfer process). All these de-excitation processes produce emission from both Nd^3+^ and Yb^3+^ ions in the NIR region (see spectra in Figure 4b).

Thus, in Figure 7a, the average efficiency of KYF:Er,Yb@KYF:Nd,Yb nanoparticle suspensions is reported as a function of the NPs concentration. Reasonably, the luminescence efficiency decreases by decreasing the nanoparticles amount. Interestingly, the minimum concentration of the NPs for which a good signal-to-noise ratio permits a reliable *in vitro* imaging is around 6 mg/mL (1:8 dilution, see Figure 7a). These luminescence properties are particularly relevant since the acquisition setup was optimized for *in vivo* imaging experiments, assessing the feasibility of these nanomaterials with a real experiment facility.

On the other hand, MRI is a powerful and well-assessed diagnostic tool that allows for imaging of tissues without the need of ionizing radiations and without any limit of penetration depth. In particular, MRI contrast agents operate by shortening the relaxation rates, *T*, of protons in the environment, and depending on the type of contrast they provide, they are called *T*_1_ (positive) or *T*_2_ (negative) contrast agents.

Nowadays, only small gadolinium-based organic molecules have been FDA-approved as positive contrast agents for nanomedicine.^56^ However, they suffer from several disadvantages, like potential biotoxicity, fast clearance from the body and thus short circulation times, as well as undesired accumulation of gadolinium ions in human tissues.^57^ To overcome these drawbacks, it is possible to incorporate paramagnetic ions inside size-tunable nanoparticles, exhibiting lower toxicity and increased retention within the body.^58^

For these reasons, the core@shell nanoparticles doped with Gd^3+^ were placed in a 7 T (7T) magnetic field and their ability to modify relaxation time of water molecules in the surroundings was investigated. Aqueous dispersions of the Gd^3+^-doped KY_3_F_10_ were evaluated at room temperature at different dilutions. It is to be noted that the Gd^3+^ concentration of the most concentrated NPs dispersion has been previously determined by ICP-MS analysis for the precise determination of its molarity. The *r*_1_ value therefore determined for the KYF:Er,Yb@KYF:Gd NPs was 1.45 ± 0.06 mM^–1^ s^–1^ (see Figure 7b), while for standard contrast agent Gadovist the reported *r*_1_ value at 7 T is 4.58 mM^–1^ s^–1^.^58^ The *T*_2_ contrast properties of the heavier paramagnetic lanthanides (Dy^3+^, Ho^3+^, Er^3+^, Tm^3+^, and Yb^3+^) occur through the Curie spin relaxation mechanism, which becomes significant at high magnetic field strengths, such as 7 T.^59,60^ Therefore, the value of *r*_2_ was also evaluated for the same NPs dispersions, for which a value of 9.49 ± 0.60 mM^–1^ s^–1^ was obtained, as shown in Figure 7c. The relaxivity values obtained in the present study are slightly different with respect to those found for other fluoride nanoparticles, namely, GdF_3_:Er,Yb^61^ (*r*_1_ = 0.02 mM^–1^ s^–1^ and *r*_2_ = 15.8 mM^–1^ s^–1^ at 4.7 T) and for a 10% Gd^3+^-doped NaYF_4_^62^ (*r*_1_ = 0.14 mM^–1^ s^–1^ and *r*_2_ = 8.7 mM^–1^ s^–1^ at 9.4 T). These slight differences arise from the fact that directly comparing the *r*_1_ and *r*_2_ values for different Gd-doped or Gd-based NPs is not trivial, since the calculated values are strongly affected by the measurement conditions (e.g., temperature, magnetic field) as well as, importantly, by the distribution of the Gd^3+^ ions on the NPs surface that are in direct contact with the water molecules, changing the proton relaxations. However, the obtained relaxivity values are significantly high to permit reliable use in biological systems as MRI contrast agents, as also described below.

Among the diagnostic techniques, computed tomography (CT) is one of the fastest, cost-effective, and deep-penetrating tools.^63^ The contrast of CT images depends heavily on the density and the atomic number of the material because it relies on the different attenuation of transmitted X-rays from the sample. While CT is excellent for imaging bones, calcified tissues, lungs, and other dense biological structures, it is poorly efficient for the visualization of soft tissues, since they suffer from very small differences in attenuation of X-rays. Thus, dense nanoparticles with a high payload offer the possibility to increase the CT contrast within soft tissues, as long as they show also excellent biocompatibility and good stability within biological environment. CT attenuation is expressed in Hounsfield units (HU),^63^ and they are usually reported in comparison with attenuation value of water (0 HU) or bones (between 400 and 1000 HU), while soft tissues range between 40 and 80 HU (see Table T6 in the Supporting Information).

Ln^3+^-doped KYF nanoparticles show very promising capability of acting as CT contrast agents in an *in vitro* experiment, both at 40 and 80 kVp of tube voltage. This is surely due to the notable attenuation of the Y^3+^ ions that are massive constituents of the host as well as to the attenuation of the lanthanide ions,^64^ in remarkably high content in the nanoparticles. Actually, Y^3+^ has a K-edge value of around 17040 eV,^65^ perfectly in the range of routinely used clinical X-rays. As reported in Figure 7d, CT attenuation capability has been investigated using a suitable standardized CT phantom^66^ for the evaluation of X-rays attenuation of NPs dispersions immersed in a tissue-simulating absorbing environment. From the CT outcome, the slopes of the linear relations between the HU values and the concentrations measured at 40 kVp for the three NPs samples are calculated and result quite similar. The small differences can be attributed to the variation in Y^3+^ content within the nanomaterials, since at low X-rays energies, only Y^3+^ has an effect on the attenuation of the signal, while the lanthanides, having higher K-edge energies, have a contribution only at a higher kilovoltage peak. It is worth mentioning the fact that the investigated nanoparticle dispersions present satisfactory HU values both at low and high tube voltages (see Table T6 and Figure S20). Moreover, it has been previously reported that the commercial X-rays CA (i.e., iopromide) shows a value of less than 40 HU@65 kVp for a concentration of 3 mg/mL.^67^ In our case, considering a nanoparticle concentration of 3 mg/mL, we obtain a contrast performance ranging from 30 to 40 HU, evaluated using the graph in Figure 7d, comparable with the clinically used compound, but, advantageously, at a lower value, 40 kVp, of the tube voltage. All these aspects contribute to the great usefulness and versatility of such nanomaterials, also because lower voltages are correlated to a lower radiation dose and are highly desirable in the case of young patients or in patients with chronic diseases, requiring frequent CT analysis. On the other hand, a high tube voltage is necessary to obtain good images with deeper body penetration.^68^ Assessing the capability to act as CT contrast agents for the present core@shell nanostructures put the basis for their use in clinical practice, also in combination with other imaging techniques, as an alternative to the iodinated molecules, suffering from several disadvantages like low payloads (few atoms for each molecule), short circulation time, and renal damage in patients with kidney diseases.^63^

#### Imaging in Biological Environments

It is widely accepted that the effect of newly discovered diagnostic or therapeutic agents on a living organism is determined by a multiplicity of parameters depending on size, morphology, nature of the surface, and its net charge. Therefore, before approaching the important but also ethically challenging field of *in vivo* experiments, we first evaluated the performance of these nanostructures as contrast agents for optical and magnetic resonance imaging, in biological tissues, namely, chicken breast.

**Figure 8:**
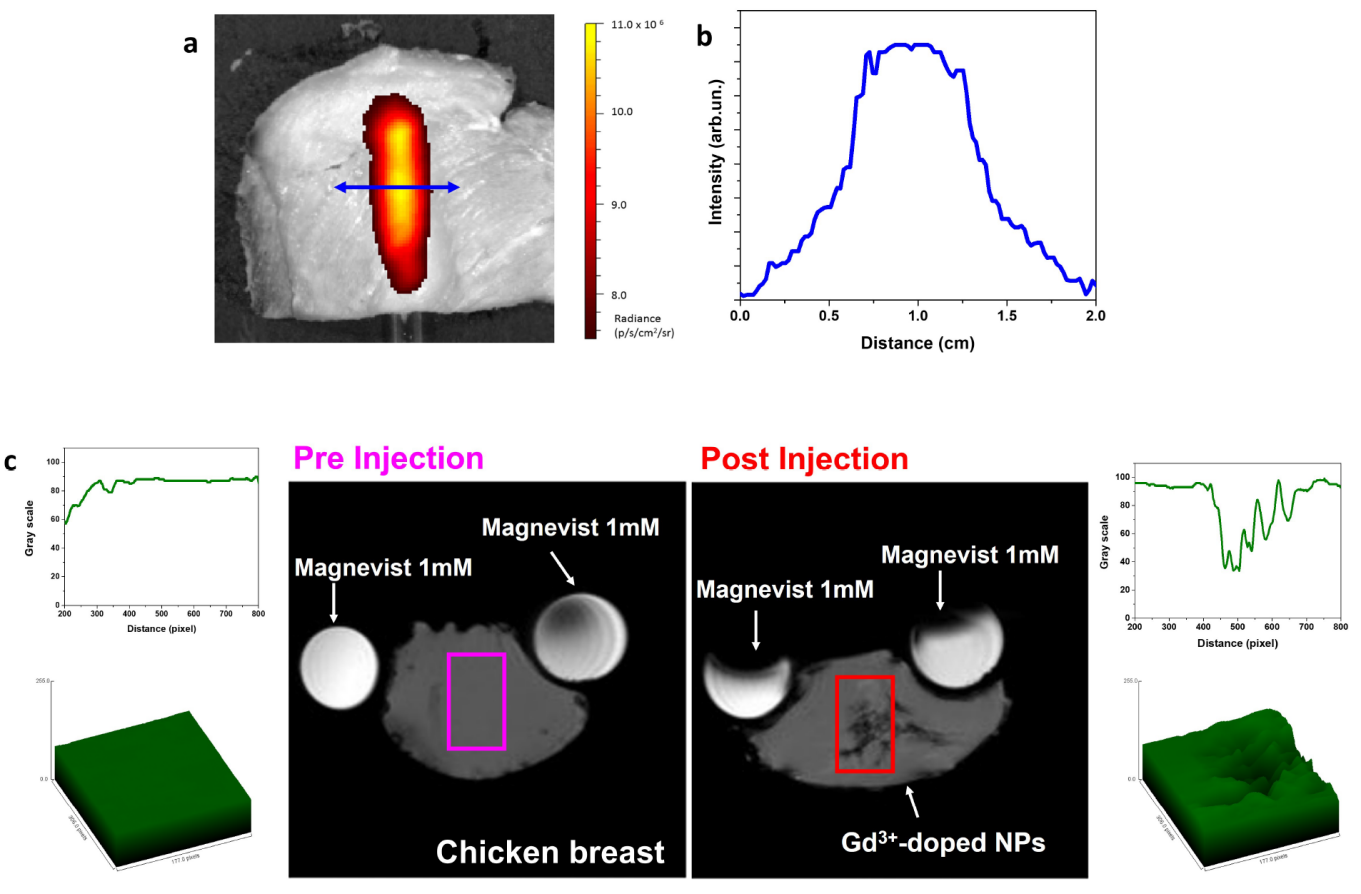
(a) Optical imaging of colloidal dispersion of KYF:Er,Yb@KYF:Nd,Yb nanoparticles (50 mg/mL) in a NMR tube, covered by a chicken breast slab (thickness of 4 mm), and (b) profile of the radiance evaluated perpendicularly to the NMR tube. (c) *T*_2_-weighted MRI of KYF:Er,Yb@KYF:Gd nanoparticles injected in a slice of chicken breast in comparison with commercial Magnevist solution.

Among the major drawbacks in using OI as diagnostic tool, the normal autofluorescence of biological components and the poor penetration depth of visible light are the most problematic. For this reason, chicken breast has been considered as illustrative biological tissue, useful as absorbing medium in the optical range for evaluation of the ability of the excitation and emission radiation to penetrate a real tissue. Upon a 570 nm excitation radiation, the NIR luminescence in the 850–1000 nm NIR range for a colloidal dispersion of the KYF:Er,Yb@KYF:Nd,Yb sample (50 mg/mL) contained in a NMR tube is clearly visible in Figure 8a, with a high emission intensity with respect to the autofluorescence of the animal tissue, precisely, a 12-fold increase of the signal-to-noise ratio. Moreover, the emission intensity profile (evaluated on a 3 pixel width line) measured perpendicularly with respect to the NMR tube, as shown in Figure 8b, reveals a very good spatial resolution around 1 mm, suggesting the possibility of applications of the luminescent core@shell nanoparticles for visualizing details of real biological structures or organs with millimetric resolution. Interestingly, the NIR emission is clearly visible with a thickness of the absorbing tissue of 4 mm, which is comparable to the thickness of the adult human skin, assessing the possibility of employing such Nd^3+^-doped nanoparticles for subcutaneous imaging.

Not only is optical imaging influenced by the absorption of surrounding environment but also the MRI technique is strongly affected by the type of tissue properties and type of acquisition parameters. In order to evaluate Gd^3+^-doped core@shell nanoparticles as magnetic contrast agents in a biological medium, namely, in chicken breast, the colloidal dispersion has been directly injected into the phantom tissue. In general, physical contact between Gd^3+^ ions and water molecules is the dominant contribution to the change in the spin–lattice relaxation time (i.e., *T*_1_), but not necessarily.^69^ In fact, contrast agents containing gadolinium shorten the *T*_1_ (or longitudinal) and *T*_2_ (or transverse) relaxation times of neighboring water protons. These effects increase the signal intensity of *T*_1_-weighted images (positive contrast) and reduce the signal intensity of *T*_2_-weighted images (negative contrast).^70^ Compared to the preinjection acquisition (left part of Figure 8c), the *T*_2_ image obtained after the injection of colloidal nanoparticles in the tissue shows a definite negative contrast, clearly visible in the 2D plot and 3D image (post injection, right part of Figure 8c), in correspondence of the injection site. In fact, Gd-doped nanoparticles can act both as *T*_1_ or *T*_2_ contrast agents, according to the type of interactions between water molecules and paramagnetic ions, to the surrounding media, to the measurement parameters, and to the presence of other paramagnetic ions, such as Dy^3+^, Ho^3+^, Er^3+^, Tm^3+^, and Yb^3+^, exhibiting a main effect on *T*_2_-weighted MRI.^60^ Thus, we have demonstrated that our Gd-doped NPs present effective *T*_2_ contrast behavior in MRI acquisition in a real biological tissue.

It has to be remarked that *T*_2_ shortening occurs at high gadolinium concentrations, which is usually of limited clinical use due to the increased risk of toxicity,^70^ especially in the case of molecular CAs like Gadovist. However, our data on cell viability after NPs incubation have proven their safety also at high concentration (500 μg/mL) in certain cell lines, and also the related high stability of the nanocrystals, demonstrated in the present study, could help avoiding the release of Gd-ions in the surrounding environment.

**Figure 9:**
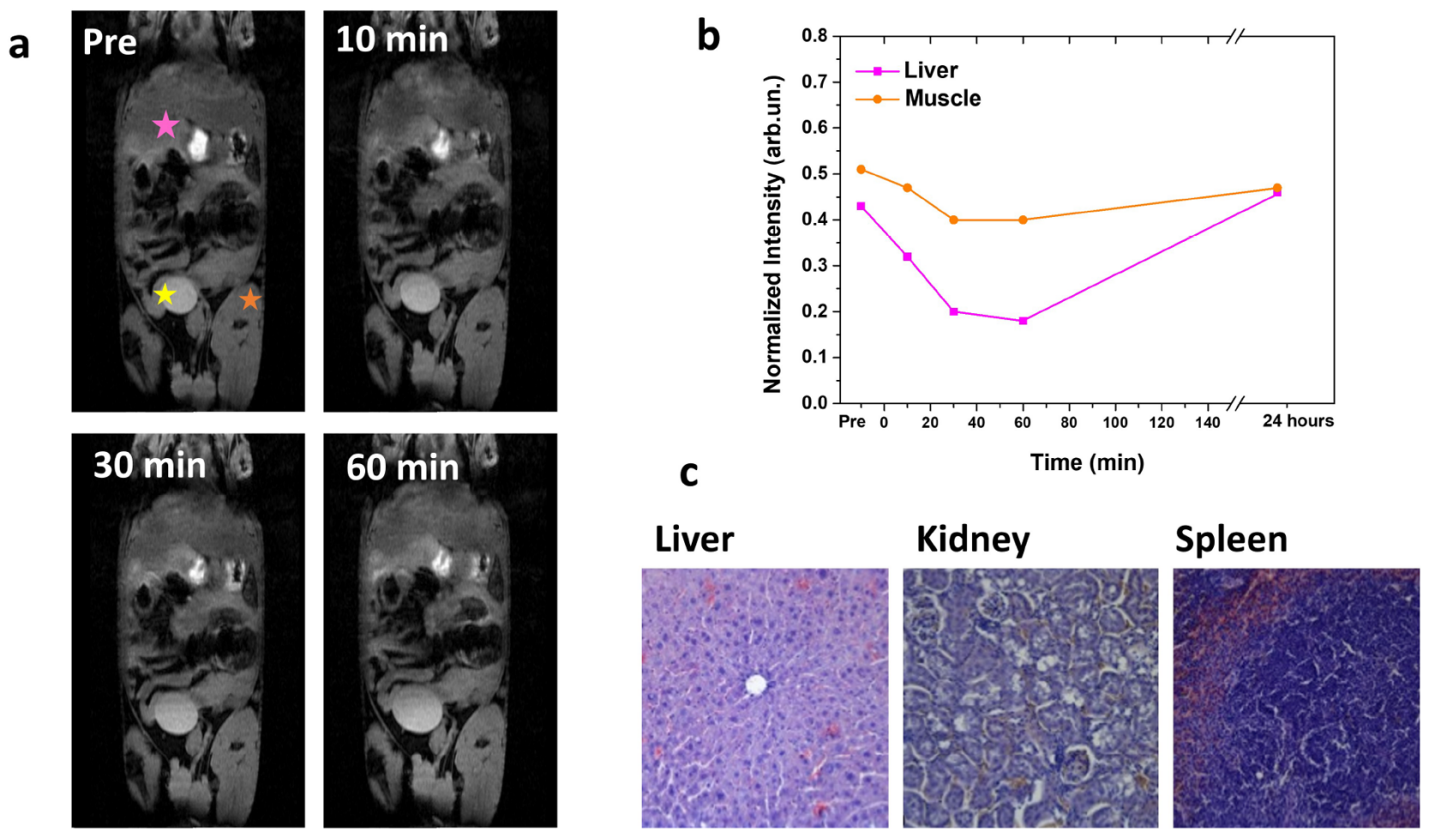
(a) *In vivo* MRI a representative animal acquired using a *T*_2_-weighted sequence before (*t* = 0) and 10 min, 30 min, and 1 h after injection of NPs dispersion. (b) T_2_-weighted signal intensity in the liver is quantitatively compared to the signal in muscle. Intensities have been normalized using a reference sample. (c) Hematoxylin and eosin staining of liver, kidney and spleen, taken 72 h after the administration of NPs, under 10× magnification with an optical microscope in bright field mode.

Finally, to demonstrate capability of our nanoparticles to be useful as multifunctional contrast agents for *in vivo* imaging, MRI biodistribution experiments have been performed by using Athymic Nude mice as a living model. After intravenously administration of colloidal NPs dispersion, *T*_2_-weighted images were acquired and the distribution of the contrast was registered at consecutive time-points (Figure 9a). A negative (darker) contrast localized in the liver immediately after 10, 30, and 60 min post-injection has been observed.

As it is possible to appreciate from Figure 9b, the *T*_2_ negative contrast after 60 min postinjection is 55% lower in the liver than in muscle (used as a control), corresponding to the time of maximum negative contrast observation. Thus, the accumulation of the NPs within the liver after 60 min post-injection can be confirmed, as previously reported in the literature for similar fluoride-based NPs,^61^ and the complete elimination from the body can be recognized by a total recovery of the signal after 24 h postinjection.

Additionally, after observation of no obvious toxic effect of administrated nanoparticles in a living animal model, H&E staining analysis of the main organs involved in the clearance of NPs (liver, spleen, and kidney), after 72 h from intravenous injection, showed no noticeable abnormality or tissue damage (see Figure 9c). This implies that the *in vivo* administration of the citrate-capped KYF NPs appears perfectly biocompatible.

## Conclusions

The investigation of new luminescent nanomaterials is of great interest, together with the identification of the main features for applications in nanomedicine. In the present work, we have investigated lanthanide-doped inorganic fluoride nanomaterials, prepared with a *green chemistry* and facile microwave-assisted procedure, which can be directly dispersed in water in colloidal form. Such nanomaterials are chemically stable in water and cell culture media as well as biocompatible. The investigated core@shell colloidal nanoparticles generate intense Vis and NIR luminescence and are suitable for thermometric measurements in the optical range. Estimated temperature variations for the used experimental setup revealed that thermometric parameters are completely reliable, since the maximum temperature variation induced by laser heating is negligible, suggesting the possibility of *in vivo* application of such nanothermometers. The Nd^3+^-doped NPs are suitable for NIR nanothermometry, with a broad range of applications including diagnostics accordingly to the available excitation radiation and detection setup. The presence of strong NIR emission also permits the use of such nanosystems as a low-autofluorescence tissue-penetrating contrast agent in optical imaging. Moreover, Gd^3+^-activated KYF NPs can be applied as multifunctional contrast agents, with both luminescent and paramagnetic properties, useful for OI and MRI. Finally, the presence of Yttrium and lanthanides offers the possibility of strongly attenuating X-rays and therefore being simultaneously usable in CT imaging. Promising results have been obtained in the present study by carrying out cell viability in both healthy and cancer cell lines, as well as performance tests in biological tissues and *in vivo* biodistribution. These achievements are a big stimulus to further development of efficient nanostructured multifunctional probes.

## Supporting Information Available

Discussions of experimental methods, XRD analysis and calculation of the lattice constant for a cubic system from XRD patterns, UC power dependence study with 4× and 40× microscope objectives, thermometric properties of colloidal dispersion of KYF:Er,Yb@KYF nanoparticles, and calculation of the induced heat and estimate of the temperature variation in a water sample and measure of temperature changes in a biological tissue under laser excitation at 980 nm, figures of TEM and HAADF-STEM images, relative size distribution plots, HAADF-STEM intensity profile, hydrodynamic diameter and ζ-potential, XRD patterns, UC spectra, absorption spectra, emission decays of the UC emissions, power plots of the upconversion emissions, logarithm of the *I*_517_/*I*_540_ intensity ratio, representative scheme of the experimental setup used for temperature and UC monitoring, temperature versus time scatter plot at different irradiation powers, and X-rays attenuation for colloidal dispersions, and tables of colloidal properties of core and core@shell nanoparticles measured by DLS technique, lattice constant calculation from the Bragg law for a cubic crystal system, decay times and weights for the biexponential fit of the green and red emission, thermometric parameter for the three LIR involving emissions, and CT attenuation values (PDF)
