# Supplementary material for: From Nanothermometry to Bioimaging: Lanthanide-Activated KY3F10 Nanostructures as Biocompatible Multifunctional Tools for Nanomedicine
Source: ACS Appl Mater Interfaces. 2023 Feb 24;15(9):12171–88. doi: 10.1021/acsami.2c22000 (PMC9999348; doi:10.1021/acsami.2c22000)
Supplement: Supplementary file 1 — am2c22000_si_001.pdf [file am2c22000_si_001.pdf]

Supporting Information

From Nanothermometry to Bioimaging: Lanthanide Activated KY_3F_{10} Nanostructures as Biocompatible Multifunctional Tools for Nanomedicine

Chiara Cressoni[a*], Federica Vurro[b*], Emil Milan[a], Matilde Muccilli[c], Francesco Mazzer[a], Marco Gerosa[c], Federico Boschi[c], Antonello Enrico Spinelli[d], Denis Badocco[e], Paolo Pastore[e], Natalia Fernandez Delgado[f], Miriam Herrera Collado[f], Pasquina Marzola[c*] and Adolfo Speghini[a*]

[a]*Nanomaterials Research Group, Department of Biotechnology, University of Verona, Strada le Grazie 15, 37134, Verona, Italy*

[b]*Division of Experimental Oncology, Urological Research Institute, IRCCS San Raffaele Scientific Institute, Via Olgettina 60, 20132, Milan, Italy; University Vita-Salute, IRCCS San Raffaele Scientific Institute, Via Olgettina 60, 20132, Milan, Italy*

[c]*Department of Computer Science, University of Verona, Strada le Grazie 15, 37134, Verona, Italy*

[d]*Experimental Imaging Centre, San Raffaele Scientific Institute, Via Olgettina 60, 20132, Milan, Italy*

[e]*Department of Chemical Sciences, University of Padova, Via Marzolo 1, 35122, Padova, Italy*

[f]*Department of Materials Science and Metallurgic Engineering and Inorganic Chemistry, University of Cadiz, Campus Universitario Rìo San Pedro, 11519, Puerto Real, Càdiz, Spain*

E-mail contacts: chiara.cressoni@univr.it; vurro.federica@hsr.it; pasquina.marzola@univr.it;

adolfo.speghini@univr.it

Contents: **Page Number**

Section S0 – Experimental section S-3
Section S1 – Morphology and size distribution analysis by TEM and high resolution HAADF-STEM S-8
Section S2 - Colloidal properties of KYF nanoparticles in water dispersion S-10
Section S3 - XRD analysis and calculation of the lattice constant for a cubic system from XRD patterns S-11
Section S4 - Spectroscopic characterization for core and core@shell nanostructures S-13
Section S5 - Emission decays of the UC emissions under pulsed 980 nm laser excitation for core and core@shell nanostructures S-15
Section S6 – Power dependence study with 4X and 40X microscope objectives S-16
Section S7 - Luminescence studies on core@shell nanoparticles in acqueous dispersion S-18
Section S8 - Thermometric properties of colloidal dispersion of KYF:Er,Yb@KYF nanoparticles using upconversion emission upon 980 nm excitation S-19
Section S9 - Calculation of the induced heat and estimate of the temperature variation in a water sample and measure of temperature changes in a biological tissue under laser excitation at 980 nm S-21
Section S10 - Thermometric parameters for the Visible-to-NIR thermometry of KYF:Er,Yb@KYF:Nd,Yb colloidal dispersion S-23
Section S11 – Cell viability on cancer and healthy cell lines S-24
Section S12 - X-Rays attenuation evaluated (in HU units) for colloidal dispersion of core@shell nanoparticles in a suitable phantom at different voltages S-25

Bibliography S-26

Section S0 – Experimental section

1. *Preparation of Er^{3+},Yb^{3+} doped KY_3F_{10} NPs (core)*

The KY_3F_{10} core nanoparticles doped with Yb^{3+} and Er^{3+} ions were synthesized via a microwave assisted hydrothermal treatment, using a microwave reactor (Monowave 400, Anton Paar). Briefly, proper volumes of aqueous solutions (1 M) of yttrium and lanthanide chlorides ($YCl_3·6H_2O$, Strem Chemicals 99.9%, $ErCl_3·6H_2O$, Strem Chemicals 99.9%, $YbCl_3·6H_2O$, Acros organics 99.99+%) in order to obtain a metal molar ratio $Y^{3+}/Yb^{3+}/Er^{3+}$= 0.78/0.2/0.02, were mixed with 13 ml of deionized water (total amount of 2.1 mmol). Then, 12 mmol of solid $K_3C_6H_5O_7·H_2O$ (potassium citrate monohydrate, Alfa Aesar 99+%), were added as powder to the previous metal precursor solution. The potassium citrate monohydrate is the source for both the potassium ions and the citrate moiety. The citrate group acts as a chelating agent for the metal ions and, after nucleation, as a capping agent on the nanoparticle surface. Then, an ammonium fluoride (NH_4F, Sigma Aldrich > 98%) aqueous solution (1.5 ml, 3.5 M) was added to the previous mixture. The obtained transparent solution was then placed in a 30 ml glass vial (G30, Anton Paar) and heat treated in the above mentioned microwave reactor at 195°C for 10 minutes. After washing with acetone, the synthesis products were redispersed in deionized water for further characterization or for growing an additional shell. The obtained nanoparticles remained stable over several months, no precipitation was detected and therefore the colloidal properties were fully maintained. The Er^{3+}, Yb^{3+} doped core KY_3F_{10} NPs are denoted in the paper as KYF:Er,Yb.

2. *Preparation of KY_3F_{10}:Er,Yb@ KY_3F_{10} NPs*

The core@shell nanoparticles were prepared using the KY_3F_{10}:Er,Yb cores as seeds, by using the following procedure. First, 4 mmol of solid potassium citrate were dissolved in 3.9 ml of deionized water in a glass vial (G10, Anton Paar). Then, a proper volume of a solution of yttrium chloride (0.7 ml, 1 M) was added, and then a total amount of 0.7 mmol of Y3+ cations were added for the shell formation. To this solution, 1 ml of a colloidal dispersion of KYF:Er,Yb NPs (concentration of 100 mg ml^{-1}) was added. Finally, 1.7 mmol of NH_4F (0.5 ml, 3.5M) were added to the resultant dispersion in the glass vial and then the vial was heat treated in the microwave reactor, at 195°C for 10 minutes. The obtained core@shell nanoparticles were precipitated and then washed with acetone and redispersed in deionized water (with a typical concentration of 20 mg ml^{-1}) for further characterizations.

The KY_3F_{10}:Er,Yb@KY_3F_{10} NPs are denoted in the paper as KYF:Er,Yb@KYF.

3. *Preparation of KY_3F_{10}:Er,Yb @ KY_3F_{10}:Gd NPs*

These core@shell NPs were prepared using the KYF:Er,Yb core NPs as seeds. After addition of 4 mmol of solid potassium citrate in 3.9 ml of deionized water contained in a glass vial (G10, Anton Paar), proper volumes of solutions of YCl_3 (0.56 ml, 1 M) and $GdCl_3$ (0.14 ml, 1 M) were added in the vial to obtain a metal molar ratio Y^{3+}/Gd^{3+}= 0.80/0.20, precursor solution to grow the MRI-active shell. Then, 1 ml of a dispersion of KYF:Er,Yb NPs (mass concentration of 100 mg ml^{-1}) was mixed to the shell precursor solution and then 1.7 mmol of NH_4F (0.5 ml, 3.5M) were added in the same vial. The resultant dispersion was heat treated in microwave reactor at 195°C for 10 minutes. The obtained core@shell NPs were precipitated and washed with acetone and redispersed in deionized water (with a typical concentration of 20 mg ml^{-1}) for further characterization.

The KY_3F_{10}:Er,Yb@KY_3F_{10}:Gd NPs are denoted in the paper as KYF:Er,Yb@KYF:Gd.

4. *Preparation of KY_3F_{10}:Er,Yb@ KY_3F_{10}:Nd,Yb NPs*

These core@shell NPs were prepared using the KYF:Er,Yb cores as seeds, as previously described. After addition of 4 mmol of solid potassium citrate in 3.9 ml of deionized water in a glass vial (G10, Anton Paar), proper volumes of YCl_3 (0.546 ml, 1M), $NdCl_3$ (0.105 ml, 1M) and $YbCl_3$ (0.049 ml, 1M) solutions were added, in order to obtain a nominal molar ratio of $Y^{3+}/Nd^{3+}/Yb^{3+}$=0.78/0.15/0.07 for the IR-active shell. Then, 1 ml of

the dispersion of KYF:Er,Yb core NPs (mass concentration of 100 mg ml^{-1}) was added to the shell precursor solution. Subsequently, 1.7 mmol of NH_4F (0.5 ml, 3.5M) were added to this dispersion and heat treated in the microwave reactor at 195°C for 10 minutes. The obtained core@shell NPs were precipitated and washed with acetone and redispersed in deionized water (with a typical concentration of 20 mg ml^{-1}) for further characterization. The KY_3F_{10}:Er,Yb@KY_3F_{10}:Nd,Yb NPs are denoted in the paper as KYF:Er,Yb@KYF:Nd,Yb.

5. *Preparation of α-$NaYF_4$:Er^{3+},Yb^{3+}@$NaYF_4$ nanoparticles*

The $NaYF_4$ core nanoparticles doped with Yb^{3+} and Er^{3+} ions were synthesized via a microwave assisted hydrothermal treatment, using a microwave reactor (Monowave 400, Anton Paar). Briefly, proper volumes of aqueous solutions (1 M) of yttrium and lanthanide chlorides ($YCl_3·6H_2O$, Strem Chemicals 99.9%, $ErCl_3·6H_2O$, Strem Chemicals 99.9%, $YbCl_3·6H_2O$, Acros organics 99.99+%) in order to obtain a metal molar ratio $Y^{3+}/Yb^{3+}/Er^{3+}$= 0.78/0.2/0.02, were mixed with 13 ml of deionized water (total metal amount of 2.6 mmol). Then, 13.3 mmol of solid $Na_3C_6H_5O_7·H_2O$ (sodium citrate dihydrate, Alfa Aesar 99+%), were added as powder to the previous metal precursor solution. Then, an ammonium fluoride (NH_4F, Sigma Aldrich > 98%) aqueous solution (3.56 ml, 3.5 M) was added to the previous mixture. The obtained transparent solution was then placed in a 30 ml glass vial (G30, Anton Paar) and heat treated in the above-mentioned microwave reactor at 170°C for 10 seconds (heating in 2 minutes). After washing with acetone, the synthesis product was stored under acetone. The synthesis of the shell was performed in the same way but using the core nanoparticles as seeds. In brief, a solution of 13.3 mmol of $Na_3C_6H_5O_7·H_2O$ (sodium citrate dihydrate, Alfa Aesar 99+%), 2.6 mmol of $YCl_3·6H_2O$ (Strem Chemicals 99.9%) and 150 mg of Yb,Er doped $NaYF_4$ NPs were mixed with ammonium fluoride (NH_4F, Sigma Aldrich > 98%) aqueous solution (3.56 ml, 3.5 M) and subjected to a temperature treated in the above-mentioned microwave reactor at 170°C for 10 seconds (heating in 2 minutes). The nanoparticles were then washed with acetone and redispersed in water for further characterization.

6. *(Scanning) Trasmission Electron Microscopy*

The nanoparticles were spread on a holey carbon grid after being dispersed into water. (S)TEM images were obtained using a Talos F200X and a FEI Titan Cubed Themis Electron Microscopes, both operating at 200 KV.

7. *Colloidal characterization*

Hydrodynamic size and ζ-potential measurements on water dispersions (mass concentration of 2 mg ml^{-1}) were performed with a Malvern Zetasizer Nano ZS operating with a HeNe 633 nm laser.

8. *X-Ray diffraction setup*

XRD measurements were carried out using a Thermo ARL X-TRA diffractometer equipped with a Copper-anode X-ray tube (Kα, λ=1.5406 Å) and a Peltier solid-stage that cools down the Si(Li) detector close to -100°C. For the measurements, the samples were dried in an oven at 80°C and homogenized in an agata mortar before deposition on a low background Si sample holder. The lattice constant for the KY_3F_{10} cubic structure was determined from the XRPD patterns and the Bragg law (see Section S3 below).

9. *Elemental analysis*

An ICP-MS instrument was used for the measurement (Agilent 7700x, Agilent Technologies International Japan, Ltd., Tokyo, Japan). The instrument is equipped with an octupole collision cell operating in kinetic energy discrimination mode, which was used for the removal of polyatomic and Ar-based interferences. The ICP-MS was tuned daily using a tuning solution containing 1 µg/L ^{140}Ce, ^{7}Li, ^{205}Tl, and ^{89}Y (Agilent Technologies, UK). The internal standard (IS) mixture (Agilent, 5183-4681) containing ^{6}Li, ^{45}Sc, ^{72}Ge, ^{103}Rh, ^{115}In, ^{159}Tb, ^{175}Lu and ^{209}Bi at 10 µg/mL each in 3.5% HNO_3 was used through addition to the sample solution via a T-junction. IS is required for both matrix and instrumental drift corrections. Samples are weighed and digested in 15 mL

Falcon (PP) tubes. Two digestion protocols were used, one with a mixture of 2.5 g of HNO_3 (69%) + 2.5 g of HCl (37%) and the other with only 2.5 g of HNO_3 (69%) both for 3h at 100°C. The digested solutions are suitably diluted to ensure that the concentrations of the elements enter the calibration line.

10. Luminescence measurements

The emission spectra were measured on the colloidal dispersion of the NPs using a laser diodes at 980 nm (MDLIII980, CNI), with variable laser power, and at 532 nm as the excitation sources. The emission signal was collected in the visible-NIR region using 4X and 40X microscope objectives and a short-pass filter with cutoff at 900 nm in the case of 980 nm laser. An half-meter monochromator (Sr-500i, ANDOR) equipped with a -80°C cooled CCD camera (iDus DU420A-BVF, ANDOR) was used as the recording setup. The optical resolution of the upconversion spectra is 5 cm^{-1}. The power density was calculated by measuring the irradiated area in the objective focal points for the two different microscope objectives (4X and 40X). For the thermometric measurements, emission spectra at different temperatures were acquired under 980 or 532 nm laser excitation, by heating the colloidal dispersions, while constantly stirring, with an aluminum heated stage, measuring the temperature with a K-type thermocouple (with a sensitivity of 0.2°C).

The decays of the UC emission were excited with the same diode laser at 980 nm, operating in TTL mode (square wave) using a waveform generator (FeelTech FY6900). The signal was collected with a 40X microscope objective and measured with the above mentioned monochromator, equipped also with GaAs photomultiplier cooled to -30°C (Hamamatsu) and a 500 MHz oscilloscope (LeCroy LT342). The emission decays were fit using the multiexponential formula:

$$I(t) = I_0 + \sum_i A_i \exp(-\frac{t_i}{\tau_i}) \qquad \text{(S1)}$$

where τ_i, A_i represent respectively the *i*-th decay time and the weight for the *i*-th component of the multiexponential function. The average lifetimes τ_{av} were calculated using the formula (Li et al., 2020)

$$\tau_{av} = \frac{\sum_i A_i \tau_i^2}{\sum_i A_i \tau_i} \qquad \text{(S2)}$$

11. Cell viability

The cytotoxicity of prepared KYF:Er,Yb@KYF, KYF:Er,Yb@KYF:Gd and KYF:Er,Yb@KYF:Nd,Yb NPs was evaluated in HEK-293, Human embryonic kidney 293 cells and MDA-MB-231 cells, human mammary carcinoma cell line, (purchased from ATCC Manassas, VA), by testing different NPs concentrations and in HLF-1, HT-29 and PANC-1 as supplementary cell lines.

HLF-1, HEK 293, PANC-1 and MDA-MB-231 cells were cultured in Dulbecco's Minimum Essential Medium (DMEM), HT-29 cells were cultured in McCoy's 5a Medium both media modified with 10% of Fetal Bovine Serum (FBS), 1% of a mix of penicillin/streptomycin 1:1 and 1% of L-glutamine 200 mM, seeded onto 96-well plates (3000 cells/well for PANC-1, HFL1, MDA-MB-231, HT-29 and 5000 cells/well for HEK 293) and incubated at 37 ◦C in humidified air with 5% CO2 for 24 h.

After 24 h, the medium was replaced with fresh medium containing increasing concentration (from 50 to 1000 µg/ml) of NPs or a fixed concentration (500 or 300 µg/ml) The MTT (3-(4,5-dimethylthiazol-2-yl) -2,5-diphenyltetrazolium bromide cytotoxicity assay was performed after 2, 24, and 48 h of incubation: 100 µl of MTT (at 5 mg/ml concentration, purchased from Sigma, Italy) were added to each well and incubated for additional 4 h (37 ◦C, 5% CO2). Formazan crystals were dissolved in 100 µl of DMSO, and the absorbance was read at a wavelength of 570 nm using a microplate reader (HTX Microplate Reader BioTek Instruments,

Winooski, VT, USA). Four measurements of optical density (OD) were recorded for each sample, and cell viability (%) was calculated with the following equation: CV(%) = ($OD_{sample}/OD_{control}$) x 100.

12. Confocal Microscopy

For confocal microscopy HEK cells were seeded on glass coverslips (200.000 cells/well), incubated for 24h with KYF:Er,Yb @ KYF, KYF:Er,Yb @ KYF:Gd and KYF:Er,Yb @ KYF:Nd,Yb NPs at the concentrations found to be non toxic according to MTT tests (150 µg/mL) . After 24 h cells were fixed with 4% (v/v) paraformaldehyde, in PBS (pH 7.4) for 30 minutes at room temperature. To visualize the intracellular distribution of nanoparticles, the cells were washed in PBS, stained for DNA with Hoechst 33342 (1µg/mL in PBS), rinsed in PBS and finally mounted in 1:1 mixture of glycerol:PBS.

For confocal microscopy, a Leica TCS SP5 AOBS system (Leica Microsystems Italia) was used: for fluorescence excitation, a diode laser at 405 nm for Hoechst 33342 and a 514 nm laser diode for NPs were employed, by collecting images in blue region (410-460 nm), in the green (520-590 nm) and in the red (610-670 nm) and by merging the bright field images for cell morphology visualization.

13. Optical Imaging

Optical images were acquired by using the IVIS Spectrum Optical Imager (Perkin Elmer, Massachusetts, USA). This instrument is equipped with a cooled (-90°C) back-thinned, back-illuminated CCD camera (active array of 1920x1920 pixels with pixel size of 13x13 µm). The optical images were acquired and analyzed with Living Image 4.5 (Perkin Elmer) software and corrected for dark measurements. A Xe-lamp and a bandpass filter centered at 570 nm (optical width of 10 nm) was used as the excitation radiation. A longpass filter with a wavelength cutoff at 850 nm (Thorlabs, New Jersey, USA) was used to collect the NIR emission, using an exposure time of 20 seconds, with a Field of View (FoV) of 13.0 cm. An NMR tube filled with NPs colloidal dispersion was covered with a chicken breast slab (thickness of 4 mm) and directly imaged with the IVIS instrument.

14. CT scanner

CT images were acquired using a small animal CT scanner (SmART, Precision X-Ray). For the measurements, two acquisition setups were used: a) 40 kVp, current of 8 mA; b) 80 kVp, current of 3 mA. Images were acquired using a 3D phantom, made by a plastic based cylindrical container, with a diameter of 70 mm and 90 mm in length, housing six 63-mm diameter polycarbonate tubes. A dataset of 360 images was acquired as a function of the measurement depth. The Hounsfield values were determined as mean values over the image dataset, by calibrating the measured ROI on the Hounsfield values for water (0 HU) and that for air (-1000 HU).

15. Magnetic Resonance Imaging

MRI was performed using a Bruker Biospin 7T Scanner (Bruker Biospin, Ettlingen, Germany). To evaluate in vitro the longitudinal and transversal relaxivities (r_1 and r_2) of KYF:Er,Yb @ KYF:Gd NPs, longitudinal and transversal relaxation times of water solutions containing different amounts of Gd were measured using a T_1map-RARE sequence with the following parameters: TR = 800.0 ms, TE = 13.3 ms, FOV = 4.20 cm, matrix size = 256 x 256, slice thickness = 1.5 mm, number of echoes = 6. Transversal relaxation rates ($1/T_1$) were plotted as a function of the Gd concentration and r_1 relaxivity was obtained by the slope of the fitting straight line. To measure the transverse relaxation time (T_2), a multislice multiecho (MSME) imaging sequence was used with the following parameters: TR = 5000.0 ms, TE = from 7.47 to 186.7 ms (number of echoes = 25), FOV = 6.00 cm, matrix size = 256 x 256, slice thickness = 1.000 mm. To evaluate the presence of KYF:Er,Yb@KYF:Gd NPs, MRI images of chicken breast were acquired using T_1w FLASH sequences with the following parameters: TR = 200.0 ms, TE = 2.8 ms, FOV = 3.00 cm, FA = 30.0 deg, matrix size = 256 x 256, slice thickness = 1.000 mm.

16. In vivo biodistribution

N=3 Athymic Nude mice (Envigo, Udine, Italy) were maintained under standard environmental conditions (temperature, humidity, and 12h/12h light/dark cycle, with water and food ad libitum) and veterinarian control in the animal facility of the University of Verona. Animal experiments were conducted following Italian law (D.L. 4 March 2014 n°26) and the European Union normative (2010/63/EU). NPs (100 µl, 20 mg ml^{-1}) were intravenously injected in healthy mice and monitored up to 24 hours, using Bruker Biospin Scanner operating at 7T (Bruker, Ettlingen, Germany). T_2 weighted images were acquired before, 10 minutes, 30 minutes, 1 hour and 24 hours after NPs injection.

17. Hystological analysis

Mice were euthanized after 72 post-injection and liver, kidneys and spleen were dissected out, washed with PBS 0.1M and fixed in 10% formalin for 4 hours. Tissues were embedded in paraffin, cut into 5µm thick sections with a microtome and dried at 37°C for 24 hours. In order to evaluate tissue morphology, sections were stained with Hematoxylin and Eosin and examined under a light microscope (Olympus BXS1) equipped with a charge-coupled device camera.

Section S1 – Morphology and size distribution analysis by TEM and high resolution HAADF-STEM

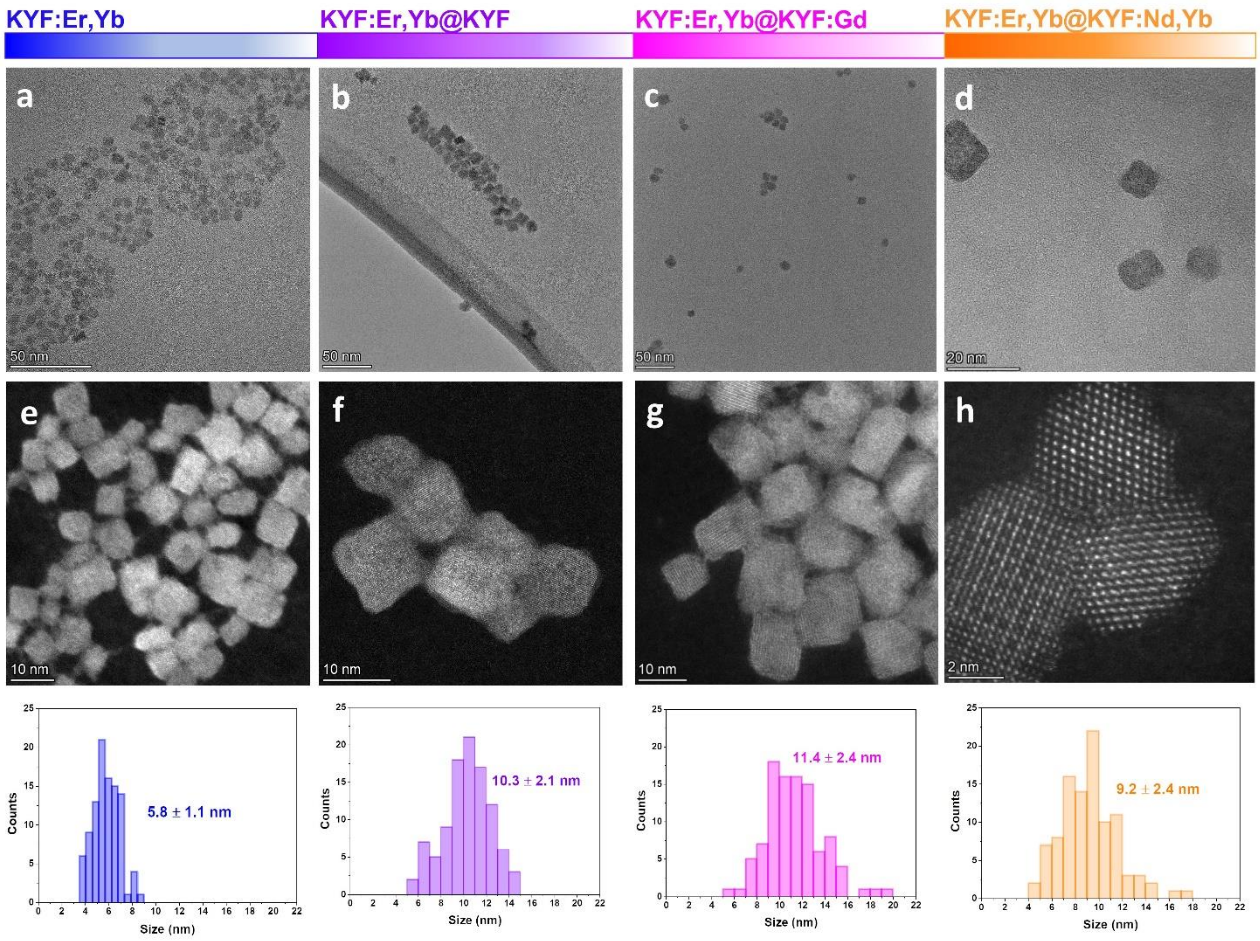

Figure S1. TEM (a-e) and HAADF-STEM images (f-g) of core and core@shell and relative size distribution plot with the average size (nm).

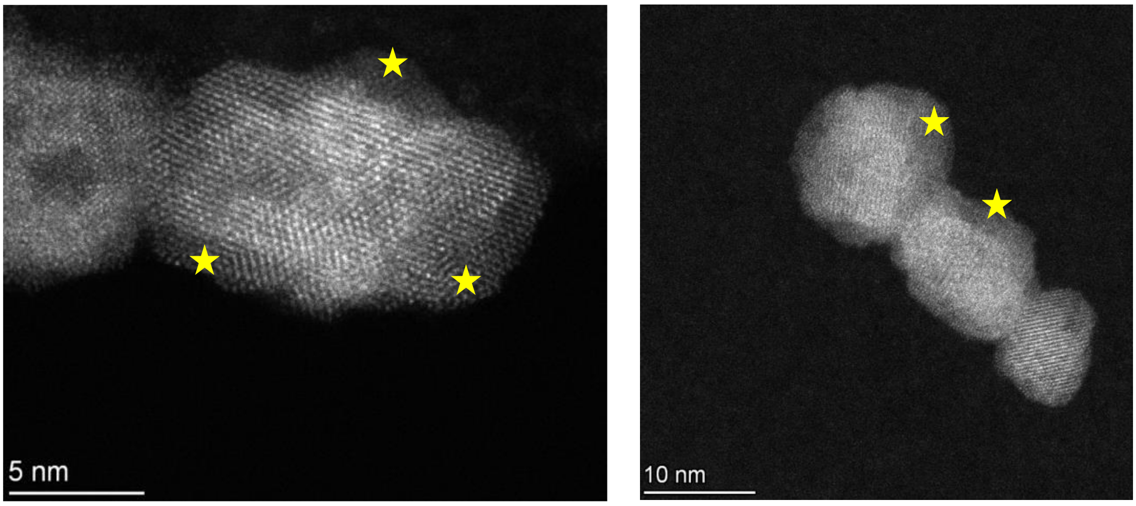

Figure S2. HAADF-STEM images of KYF:Er,Yb@KYF nanoparticles. The yellow stars represent the regions with lower HAADF-STEM intensity around the nanoparticles, suggesting the presence of the undoped shell.

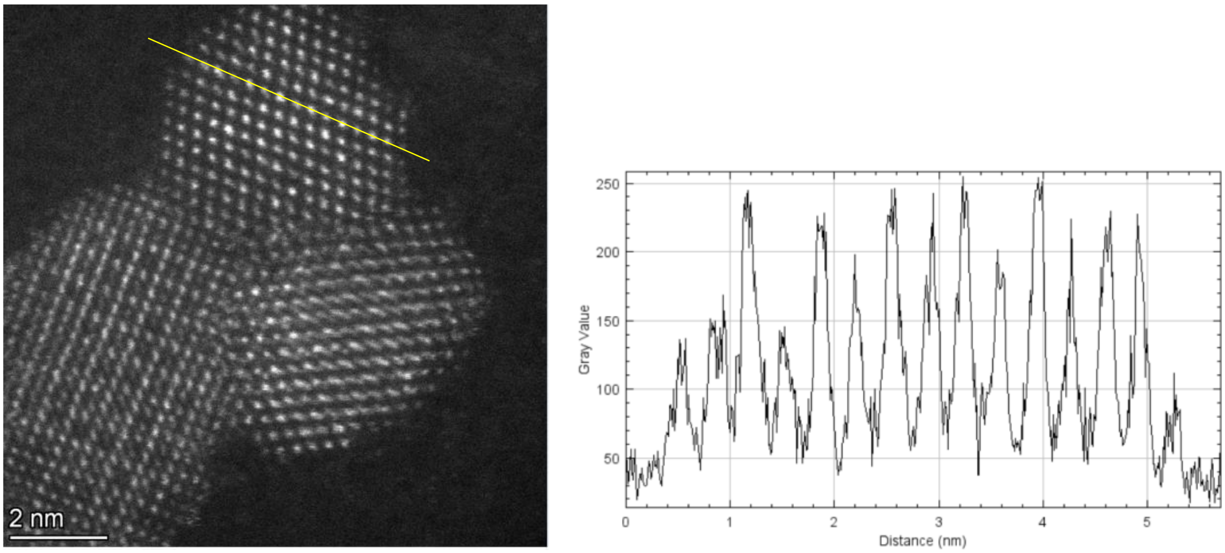

Figure S3. HAADF-STEM image of KYF:Er,Yb@KYF:Nd,Yb nanoparticles and its respective HAADF-STEM intensity profile (of yellow line).

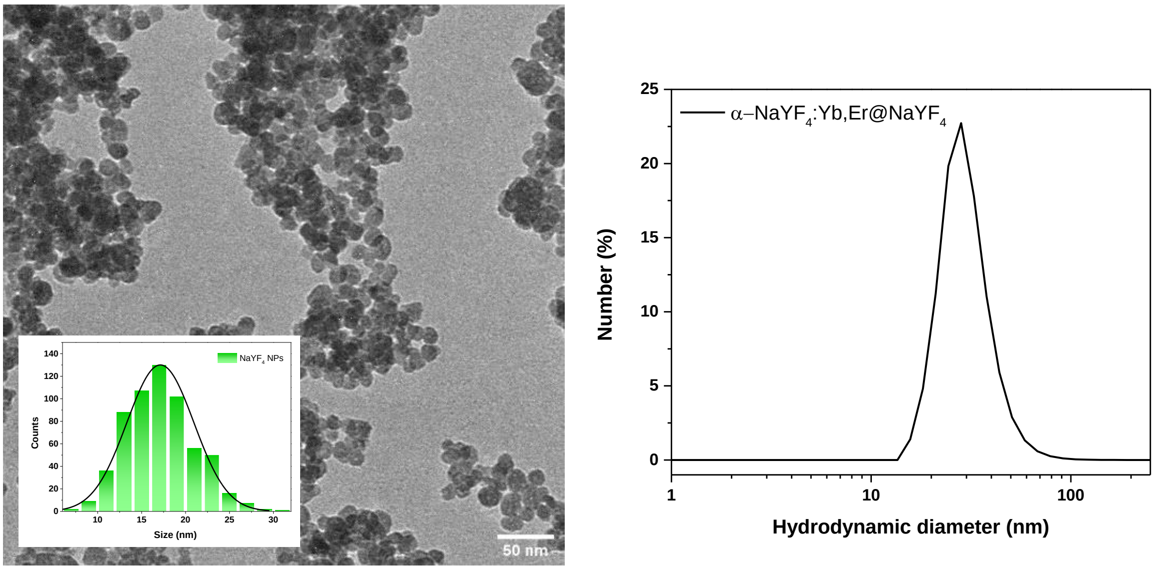

Figure S4. TEM image and relative size distribution for α-$NaYF_4$:Er,Yb (core) and particle size distribution of α-$NaYF_4$:Er,Yb@$NaYF_4$ NPs.

Section S2 - Colloidal properties of KYF nanoparticles in water dispersion

Table T1. Colloidal properties of core and core@shell nanoparticles measured by DLS technique.

Sample	Hydrodynamic diameter (nm)	PDI	ζ-potential (mV)
KYF:Er,Yb	8.6±2	0.201	-30.2 ± 9.7
KYF:Er,Yb@KYF	13±2.6	0.361	-31.5 ± 15
KYF:Er,Yb@ KYF:Nd,Yb	12.3±2.5	0.819	-36.2 ± 12.7
KYF:Er,Yb@ KYF:Gd	11.3±2.8	0.382	-32.4 ± 11.5

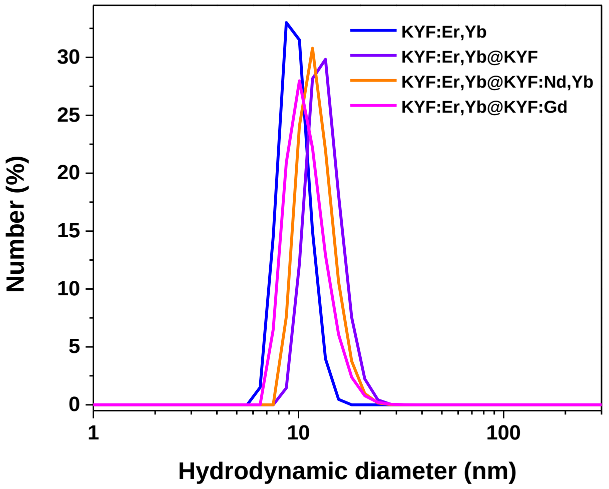

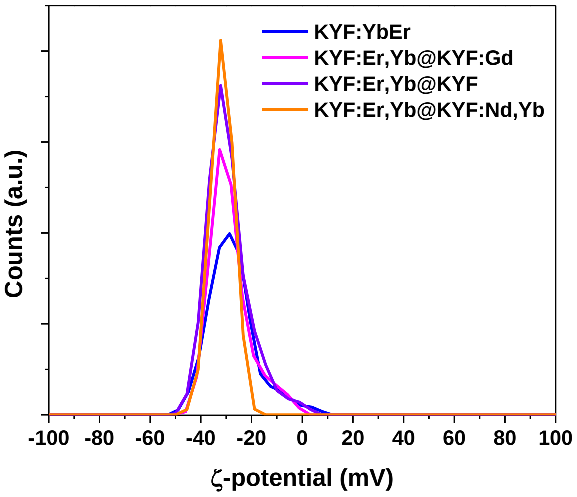

Figure S5. Hydrodynamic diameter and ζ-potential for aqueous colloidal dispersions of core and core@shell nanoparticles.

Section S3 - XRD analysis and calculation of the lattice constant for a cubic system from XRD patterns

Using the Bragg law (Hammond, 2015)

$$n\lambda = 2dsin\theta \qquad \text{(S3)}$$

Where λ is the wavelength of the X-ray beam (for Cu, 1.54 Å). The interplanar spacing d_{hkl} can be calculated and used to determine the lattice constant *a*. In case of a cubic system:

$$d_{hkl} = \frac{a}{\sqrt{h^2+k^2+l^2}} \qquad \text{(S4)}$$

where (hkl) are the Miller indices.

For the core nanoparticles four peaks were chosen for the calculation of lattice constant, after fitting each peak with a gaussian function for the determination of 2θ values in the experimental pattern.

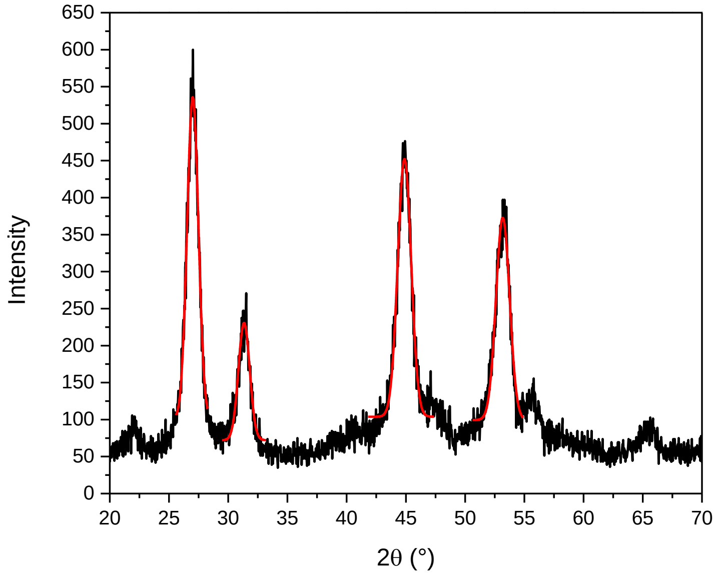

Figure S6. Experimental XRD pattern for the KYF:Er,Yb core nanoparticles (black line) and fit of the more intense reflections (red line).

Table T2. lattice constant calculation from the Bragg law for a cubic crystal system

Angle (2θ)	Miller indices (hkl)	Spacing (d_{khl})	Lattice spacing (a)
27°	(2 2 2)	3.2997 Å	11.4259 Å
31.33°	(4 0 0)	2.8537 Å	11.4148 Å
44.87°	(4 0 4)	2.0184 Å	11.4178 Å
53.18°	(2 2 6)	1.7209 Å	11.4152 Å

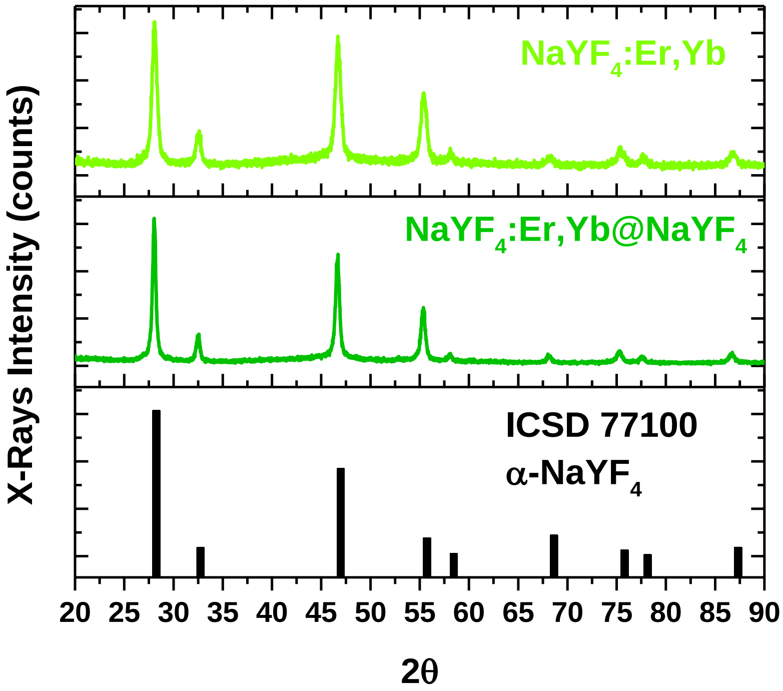

Figure S7. Experimental XRD pattern for the α-$NaYF_4$:Er,Yb core nanoparticles (light green line) and core@shell NPs (dark green line), compared to the corresponding databased pattern from ICSD.

Section S4 – Spectroscopic characterization for core and core@shell nanostructures

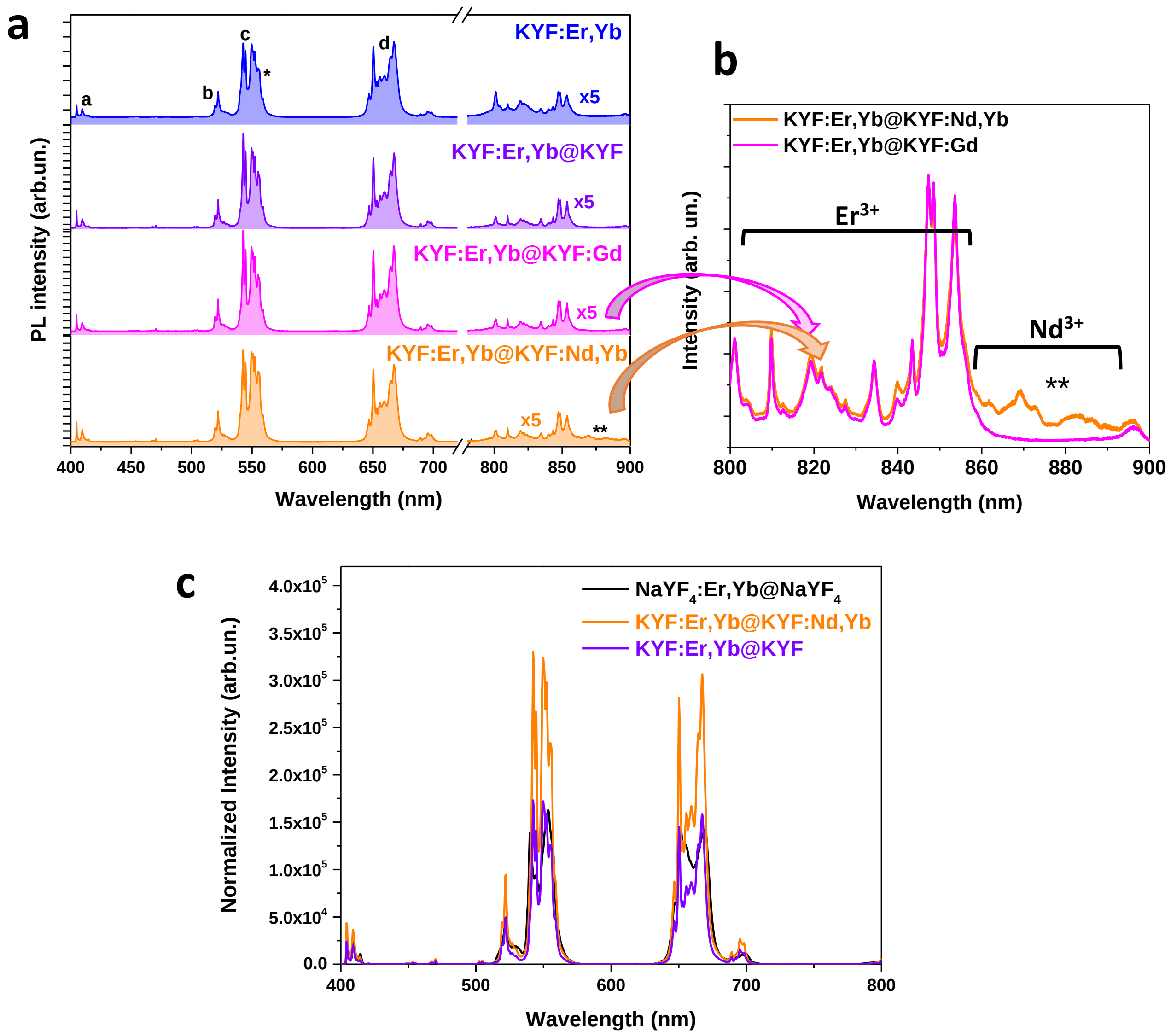

Figure S8. (a) UC spectra (λ_{exc}=980 nm) for colloidal dispersions of core and core@shell nanostructures. Assignment of the bands due to transitions of the Er^{3+} ions: (a) $^2H_{9/2} \rightarrow {}^4I_{15/2}$; (b) $^2H_{11/2} \rightarrow {}^4I_{15/2}$; (c) $^4S_{3/2} \rightarrow {}^4I_{15/2}$; (*): $^2H_{9/2} \rightarrow {}^4I_{13/2}$; (d) $^4F_{9/2} \rightarrow {}^4I_{15/2}$. (b) detail of the UC spectra for KYF:Er,Yb@KYF:Nd,Yb and KYF:Er,Yb@KYF:Gd. **: weak Nd^{3+} emission bands, due to energy transfer processes from Yb^{3+} and/or Er^{3+} ions to Nd^{3+}. (c) Comparison of UC emission spectra of KYF:Er,Yb@KYF, KYF:Er,Yb@KYF:Yb,Nd and $NaYF_4$:Er,Yb@$NaYF_4$ in colloidal dispersion, normalized on Yb^{3+} concentration, under 980 nm laser excitation.

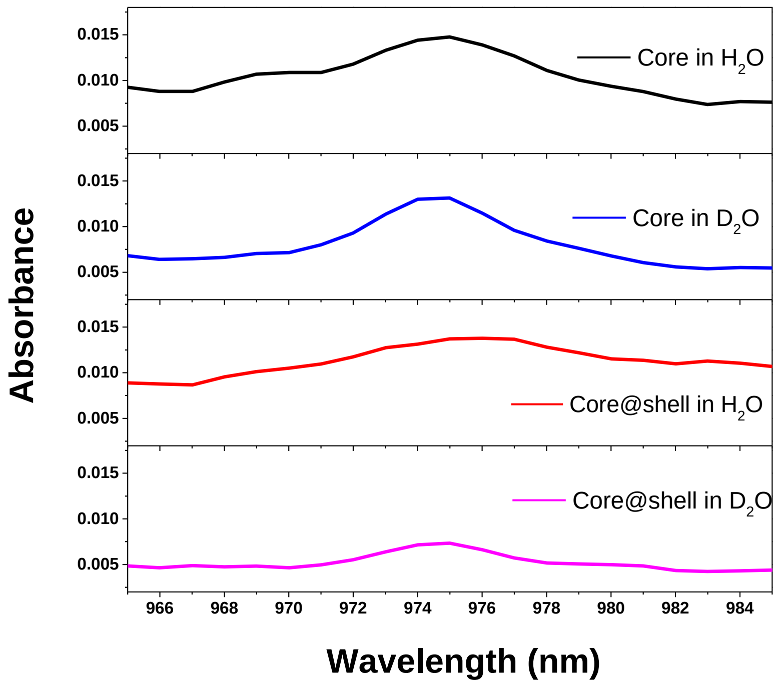

Figure S9. Absorption spectra of the KYF:Er,Yb (core) and the KYF:Er,Yb@KYF:Gd (core@shell) colloidal dispersions in H_2O and D_2O. The integrated areas of the Yb^{3+} absorption bands were used to normalize the UC spectra of the colloids, for the same solvent (H_2O or D_2O) (see Figure 3 and Figure S10).

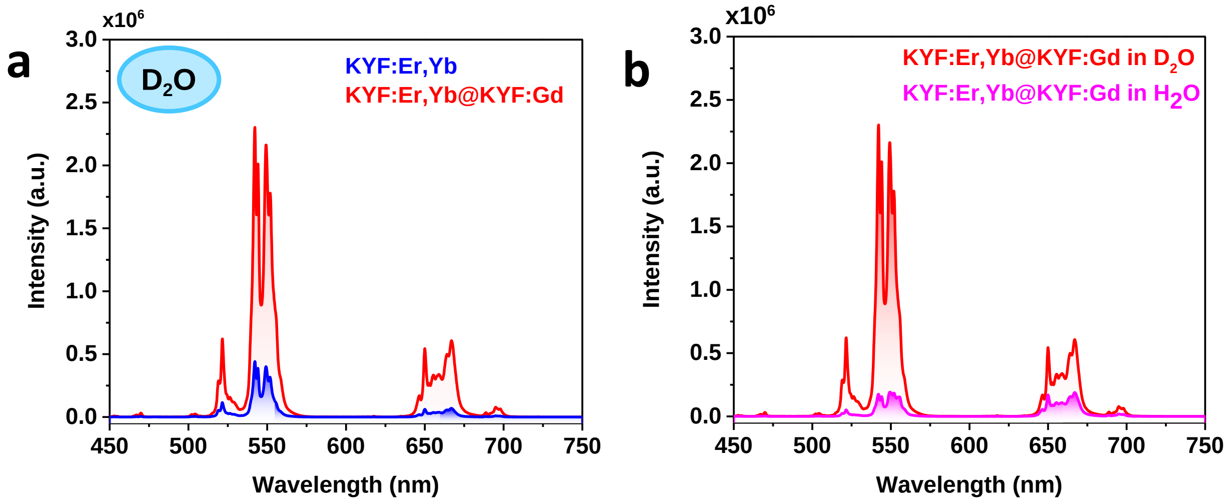

Figure S10. (a) UC spectra (λ_{exc}=980 nm) for the KYF:Er,Yb (core) and KYF:Er,Yb@KYF:Gd (core@shell) colloidal dispersions D_2O. (b) UC spectra (λ_{exc}=980 nm) for the KYF:Er,Yb@KYF:Gd (core@shell) in D_2O and H_2O.

Section S5 - Emission decays of the UC emissions under pulsed 980 nm laser excitation for core and core@shell nanostructures.

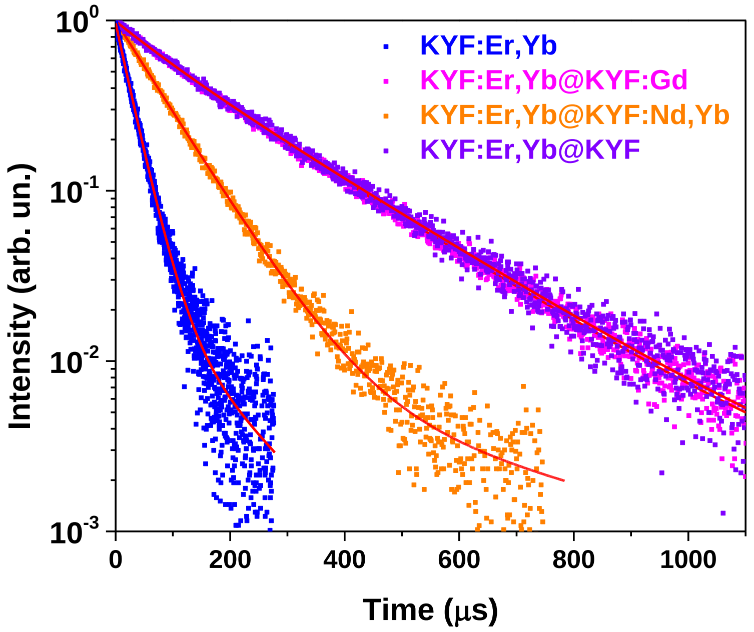

Figure S11. Emission decays (λ_{exc}=980 nm) of the red UC emission (λ_{em}=650 nm) for the core and core@shell nanostructures in H_2O colloidal dispersions (concentration of 20 mg/ml). Solid lines: fits with biexponential functions (Equation S1).

Table T3. Decay times τ_i and weights A_i for the biexponential fit of the green emission (λ_{em}=543 nm); average lifetimes τ_{av} (Equations S1 and S2)

Nanoparticles	τ_1 (μs)	A_1	τ_2(μs)	A_2	τ_{av}(μs)
KYF:Er,Yb	21.8 ± 0.2	0.91	87.4 ± 10.6	0.04	31.6
KYF:Er,Yb@KYF	65.0 ± 4.0	0.34	150.0 ± 3.8	0.66	134.5
KYF:Er,Yb@KYF:Gd	79.2 ± 3.4	0.45	157.0 ± 5.1	0.52	133.4
KYF:Er,Yb@KYF:Nd:Yb	58.2 ± 0.4	0.99	352.6 ± 42.0	0.01	75.4

Table T4. Decay times τ_i and weights A_i for the biexponential fit of the red emission (λ_{em}= 650 nm); average lifetimes τ_{av} (Equations S1 and S2)

Nanoparticles	τ_1(μs)	A_1	τ_2 (μs)	A_2	τ_{av}(μs)
KYF:Er,Yb	27.7 ± 0.2	0.93	123.3 ± 20	0.03	39.6
KYF:Er,Yb@KYF	94.7 ± 7.5	0.25	213.4 ± 4.5	0.74	197.9
KYF:Er,Yb@KYF:Gd	94.8 ± 4.9	0.26	210.8 ± 3.0	0.73	194.8
KYF:Er,Yb@KYF:Nd:Yb	80.1 ± 0.4	0.98	475.9 ± 160.0	0.01	102.7

Section S6 – UC Power dependence study with 4X and 40X microscope objectives

In order to further characterize the luminescence properties of the Ln^{3+}-doped KY_3F_{10}, two excitation power ranges have been considered for power-dependence study: in particular, a lower power density is obtained using a 4X microscope objective with wider spot-size, while a higher power density is achieved using a 40X microscope objective with smaller spot-size. The green and red UC emissions are here considered in a separate way for better explanation.

The green emission band arises from the radiative emission of three different Er^{3+} energy levels, as represented in Figure 3b in the main paper. $^2H_{11/2}$ and $^4S_{3/2}$ levels are populated through a two-photons process. On the other hand, the blue and green emissions originating from $^2H_{9/2}$ require a three photons mechanism (see Figure 3b). At low P (i.e. using the 4X microscope objective), no three photons up-conversion mechanism are detected, as shown in Figure S12. In fact, for power density around 1.2 W cm^{-2} there is no observable blue emission UC band, and the green one lacks the low energy component, around 555 nm. On the other hand, in the high power density regime, from a power density of 20 W cm^{-2}, both the blue emission band (around 400 nm) and the lower energy component of the green band are evident, pointing out that three photons absorption mechanism is active. The power dependence (log-log plot) of the green UC bands at low and high P results in slope values of 1.32 and 1.49, respectively (Figure S13, a and b). The steeper slope observed for high power densities suggests that also three photons up-conversion processes are involved in the green emission.

For what concerns the red band, it arises from the radiative emission of the $^4F_{9/2}$ energy level of the Er^{3+} ion. This energy level is populated through at least two different two-photon excitation pathways(Würth et al., 2017). The competition between the upconversion process and non-radiative relaxation is at the base of the different power dependence at low and high P for the red UC emission of Er^{3+}: at low P, depopulation of the thermally coupled green emitting levels down to $^4F_{9/2}$ level is predominant, leading to an increase of red emission. Differently, at high P, energy transfer upconversion (ETU) is the most favorite process, leading to a preferential population of the $^4F_{9/2}$ energy level and depopulation of the red emitting energy level (Pollnau et al., 2000), as clearly evidenced from the comparison of the UC spectra shown in Figure S10. This behavior not only affects the P dependence of the red band, but also explains the higher intensity of the red band in comparison to the green one at lower P (when using a 4X microscope objective). This is also confirmed by the calculated value of slopes for the power study of the red emission, resulting to be 1.32 at low P and 1.14 at high P (Figure S13, a and b). Finally, as a general consideration, the slope value n smaller than 2 can be related to different concurrent absorption and emission mechanisms, especially non-radiative de-excitation processes, cross relaxations, energy migration between active ions, all concurring in significant lowering of the experimental value of the slope (Pollnau et al., 2000).

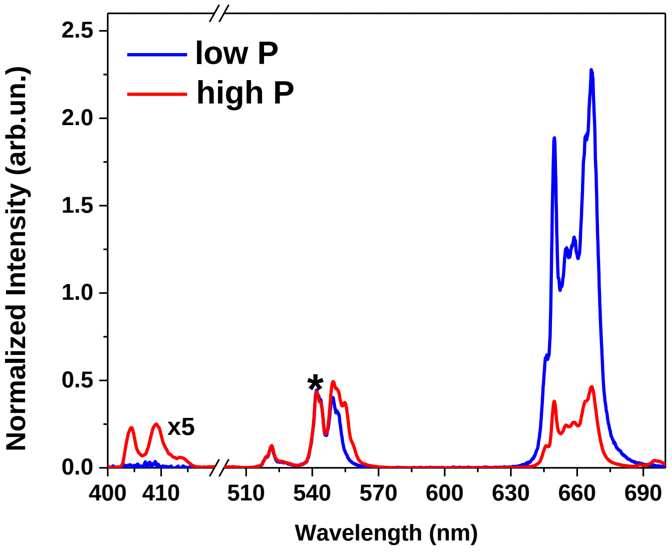

Figure S12. Upconversion spectra of the KYF:Er,Yb@KYF colloidal dispersion under 980 nm laser excitation, at low power density P (1.2 W cm^{-2}, blue line) and high P (20 W cm^{-2}, red line). The band on which the normalization is carried out is indicated with *.

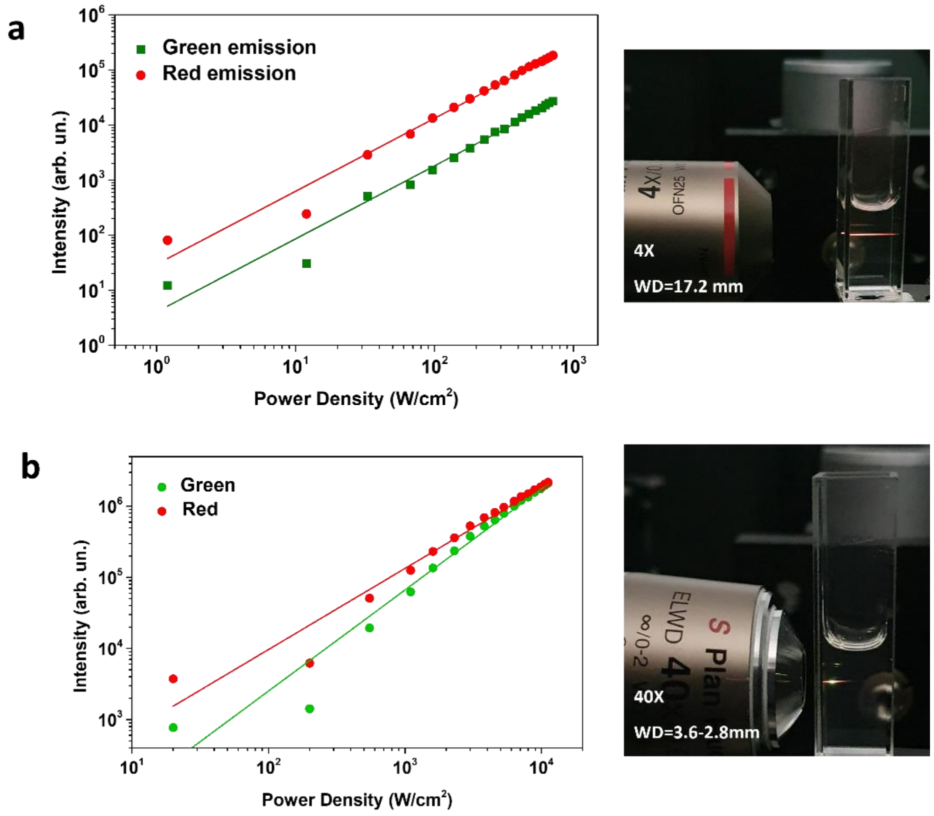

Figure S13. Power plots of the upconversion emissions (green and red emissions) for KYF:Er,Yb@KYF sample under 980 nm laser excitation and collected in backscattering mode by using a 4X microscope objective (minimum power density 1.2 W cm^{-2}) (a) and a 40X microscope objective (minimum power density 20 W cm^{-2}) (b), with the relative pictures of KYF:Er,Yb@KYF:Gd sample in acqueous colloidal dipersion under 980 nm laser irradiation, using the two different microscope objectives.

Section S7 – Luminescence studies on core@shell nanoparticles in acqueous dispersion

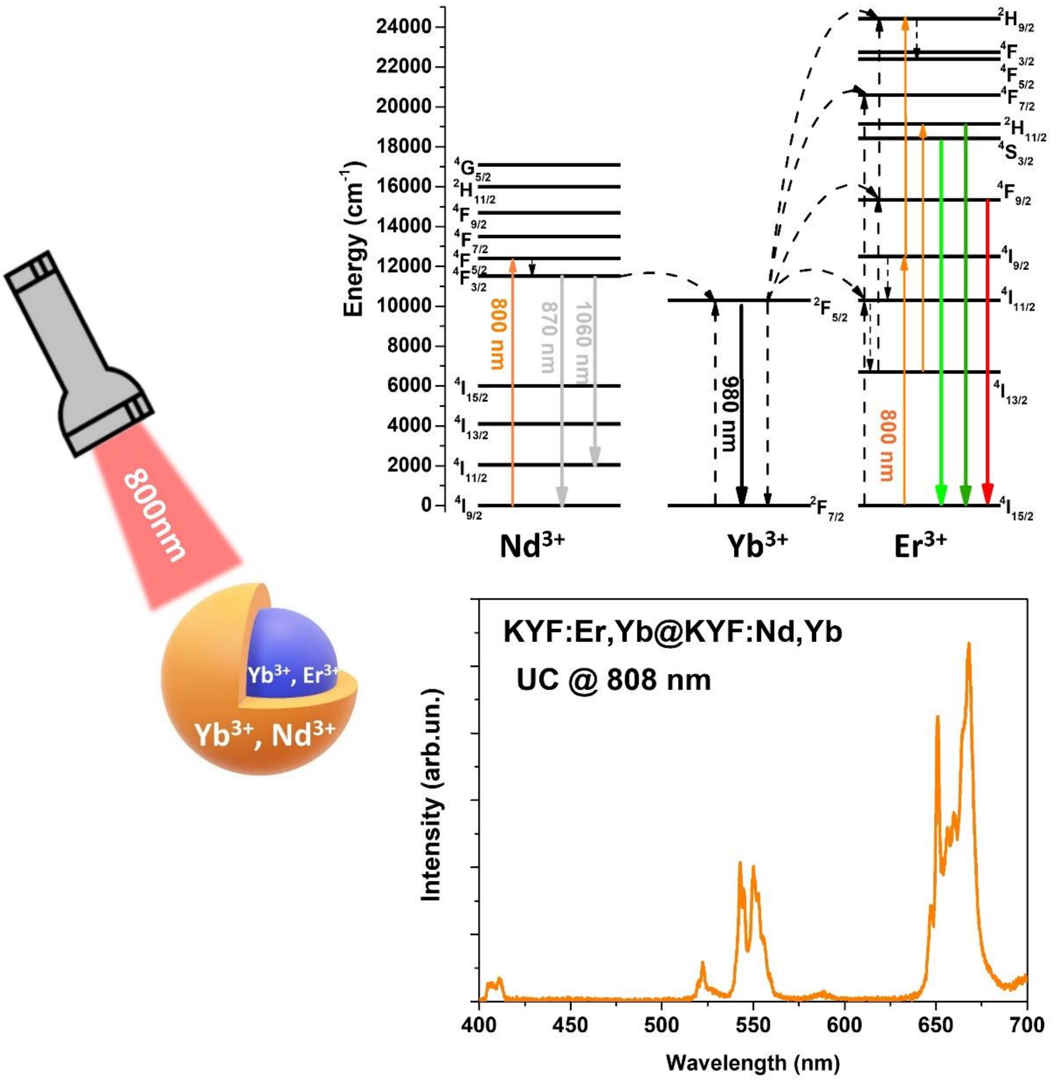

Figure S14. Upconversion spectra for KYF:Er,Yb@KYF:Nd,Yb nanoparticles under 808 nm laser excitation, with the Nd^{3+}-Er^{3+}-Yb^{3+} energy level scheme and corresponding energy transfer mechanisms.

Section S8 - Thermometric properties of colloidal dispersion of KYF:Er,Yb@KYF nanoparticles using upconversion emission upon 980 nm excitation

From the upconversion spectra under 980 nm laser excitation, thermometric measurements for the KYF:Er,Yb @ KYF nanostructures, have been performed by exploitation of thermosensitive radiative transitions $^2H_{11/2} \rightarrow {}^4I_{15/2}$ and $^4S_{3/2} \rightarrow {}^4I_{15/2}$ of the Er^{3+} ion, centered at 517 and 540 nm respectively, within the visible region (Vetrone et al., 2010).

The emission spectra in the 20-50 °C temperature range have been acquired for water colloids of core@shell nanoparticles with a concentration of 20 mg ml^{-1} (Figure S15a). The relevant thermometric parameter LIR (Luminescence Intensity Ratio, defined in Equation S6) is calculated as the ratio of the integrated intensities I_{517} and I_{540} of the two above mentioned bands, and it is shown in Figure S15b. The ln(LIR) vs 1/T curve for the Er^{3+} green emissions was fitted according to the Boltzmann distribution formula (see Figure S16):

$$LIR(Er) = \frac{I_{517}}{I_{540}} = Ae^{-\Delta E/k_B T} \quad \text{(S6)}$$

where *A, ΔE, k_B and T* are respectively the pre-exponential term, the energy gap between the two thermalized energy levels, the Boltzmann constant and the absolute temperature, respectively (Vetrone et al., 2010). From the Boltzmann fit, shown in Figure S16, the energy gap ΔE between the $^2H_{11/2}$ and $^4S_{3/2}$ levels results of 858 cm^{-1} K^{-1}, while Solanki et al. found a value around 800 cm^{-1} for the same host (Solanki et al., 2021). This small difference between the energy gaps could be related to a slightly different local environments around the lanthanide ions, on the average slightly more distorted, especially at the particle surface, in the present case, due to the smaller size and to the presence of coordinating water molecules.

The thermometric performance of the nanoparticles is evaluated by calculating the relative thermal sensitivity, defined as

$$S^{rel} = \frac{1}{LIR}\frac{dLIR}{dT} \quad \text{(S7)}$$

and the $S^{rel}(Er)$ values as a function of the temperature are shown in Figure S15c. The $S^{rel}(Er)$ values range from 0.9%K^{-1}@343 K to 1.47%K^{-1}@ 307. The maximum value is slightly higher than the value reported in Solanki et al. (Solanki et al., 2021) for the same core@shell structure (1.245%K^{-1}@300K), although the sample is analyzed in powder form of much larger nanoparticle size.

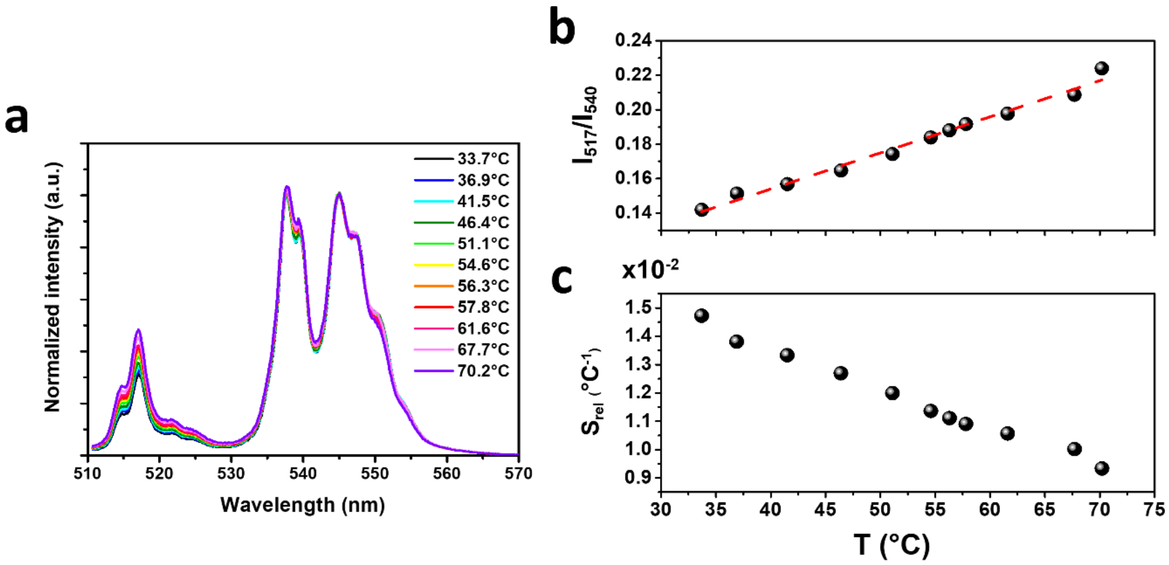

Figure S15. (a) Upconversion emission of Er^{3+}-doped core@shell KYF:Er,Yb@KYF nanoparticles under 980 nm laser excitation at different temperatures within the range 33.7-70.2°C. (b) The LIR(Er) intensity ratio vs T and (c) the relative thermal sensitivity S^{rel}(Er) vs T.

The average ΔT_{min} is evaluated to be 1.0°C, according to eq. 4, main text.

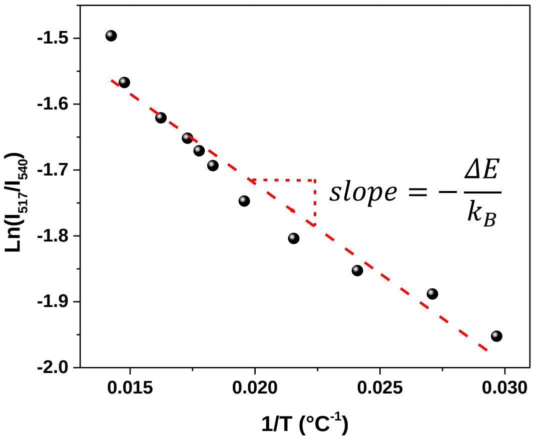

Figure S16. Logarithm of the I_{517}/I_{540} intensity ratio for KYF:Er,Yb@KYF aqueous colloids (λ_{exc}=980) vs 1/T (black dots); fit according to the Boltzmann equation, Equation S6 (dashed line, red).

Section S9 - Calculation of the induced heat and estimate of the temperature variation in a water sample and measure of temperature changes in a biological tissue under laser excitation at 980 nm

First, for evaluating the heating induced by the excitation radiation, we consider a laser beam at 980 nm wavelength hitting a water sample contained in a laboratory cuvette (4×1×1 cm), kept under constant stirring. The increase of the temperature of the water sample in these experimental conditions can be calculated assuming that the absorbed power is completely converted into heat, depending on the incident laser power and the absorption coefficient of water at 980 nm.

In particular, we consider our actual experimental conditions involved in our UC nanothermometry investigation on KYF:Er,Yb@KYF nanostructures, i.e. an incident laser power of 0.45 W and an irradiation time of 2 seconds.

We consider that the heat induced in the system Q is proportional to the absorbed power due the exciting laser radiation I_{las} and to the irradiation time Δt according to:

$$Q = I_{las} * \Delta t$$

On the other hand, the induced variation of the temperature ΔT depends on the heat capacity of the system C_s according to the:

$$Q = C_s * \Delta T$$

In our thermometry experiment (see Section S8), an incident laser power (I_0) of 0.45 W was used, with an irradiation time of 2 seconds, for measuring the UC spectra. The heat capacity of our system (assuming pure water), can be calculated from:

$$C_s = m * c_s$$

where c_s is the specific heat capacity of water, 4.18 J g^{-1}°C^{-1}, m is the mass of the sample (water).

If we consider the absorption coefficient of H_2O at 980 nm, α(980), the actual power absorbed by the sample I_{las} can be estimated according to:

$$I_{las} = I_0(1 - 10^{-\alpha l})$$

where l is the optical path or the radiation, in our case 1 cm considering a glass square cuvette and α(980)=0.482 cm^{-1}. Therefore, we obtain a value of I_{las} = 0.3 W.

Assuming a mass of water of 4 g inside the cuvette, the estimated temperature increase for a 10 seconds irradiation time, which we will consider as the maximum ΔT (ΔT_{max}) for this irradiation experiment, is $\Delta T \approx$ 0.04°C.

As a further proof of concept of the reliability of our thermometric analysis, we carried out an experiment using a biological tissue, i.e. a chicken breast slice. In particular, 20 μl of the KYF:Er,Yb@KYF colloidal dispersion (concentration of 20 mg/ml) was injected into a chicken breast slice (about 6 cm^2) and the temperature variation of the biological tissue was monitored as a function of the incident laser powers and irradiation times. It is worth noting that the UC emission is clearly visible at the naked eye (see Figure S17a). Thermal images taken with a thermal camera of the chicken slice under different irradiation powers and times are shown in Figure S17b. From the thermal pictures, the temperature values of the biological system have been extracted and they are plotted vs time in Figure S18, where the behaviors as a function of the incident laser power have been also shown. It can be clearly noted from the Figure S18 that before 50 seconds under CW 980 nm laser irradiation the maximum induced temperature variation is close to 1°C.

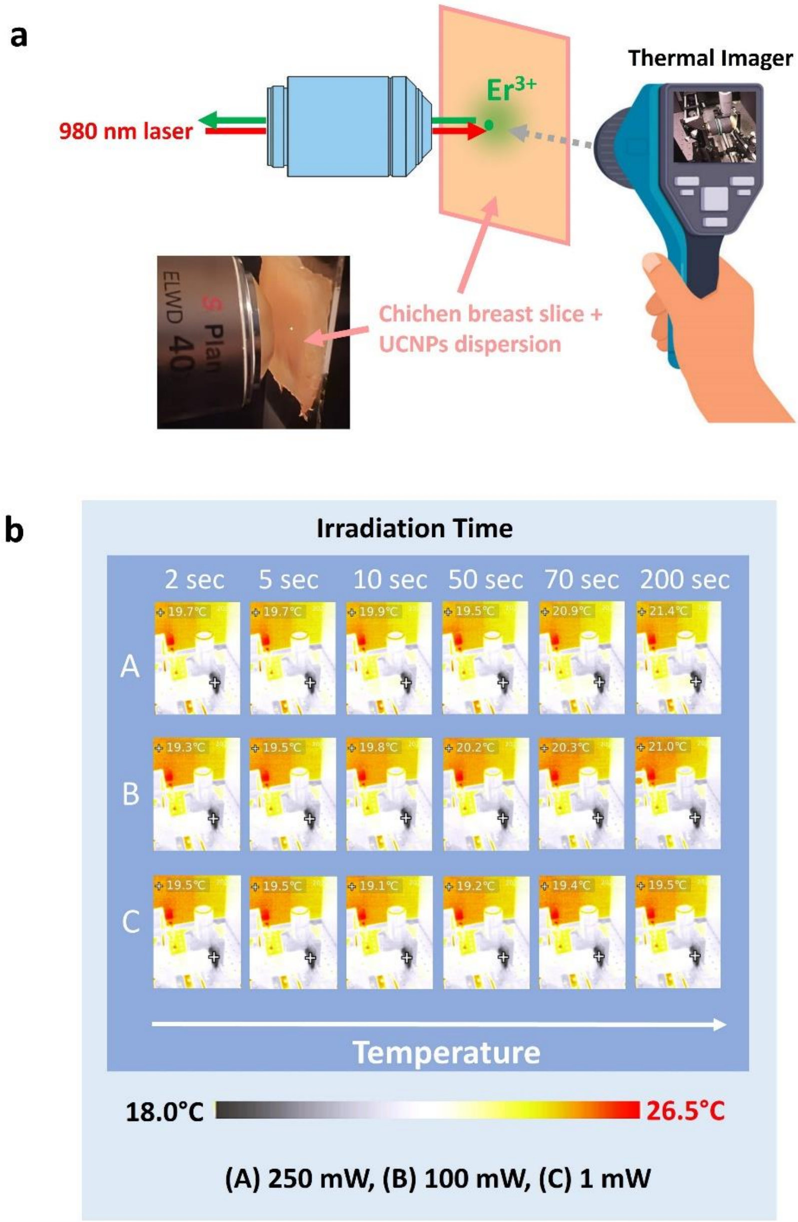

Figure S17. Representative scheme of the experimental setup used for temperature and UC monitoring on the chicken tissue (a). Thermal images taken with thermal camera of the chicken slice under different irradiation powers and times (b).

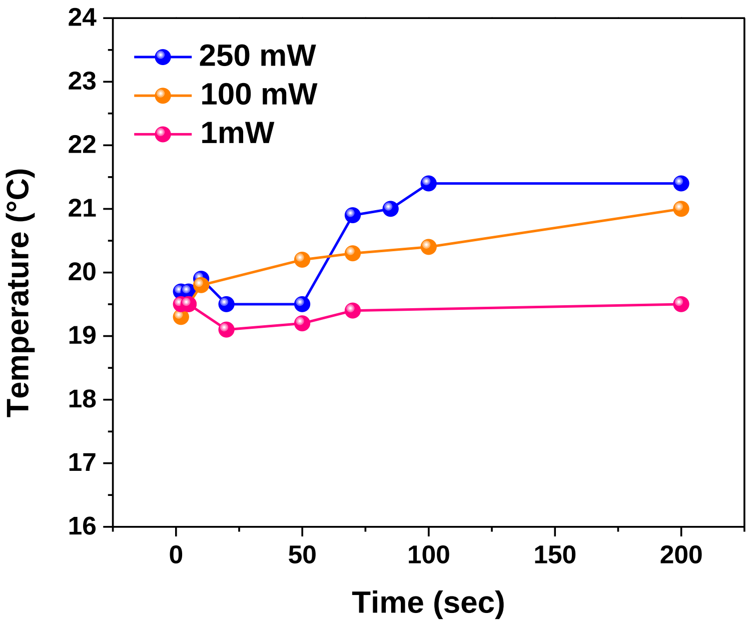

Figure S18. Temperature versus time scatter plot at different irradiation powers of the CW 980 nm laser, irradiating the chicken tissue.

Section S10 – Thermometric parameters for the Visible-to-NIR thermometry of KYF:Er,Yb@KYF:Nd,Yb colloidal dispersion

Table T5. Thermometric parameter for the three LIR involving emissions in the NIR region of the Nd^{3+} and Yb^{3+} ions.

LIR	Involved emission bands (nm)	S^{rel} @ 310 K	ΔT_{min} (K)	R (%)
LIR(Yb/Nd)	976, 862	0.57 % K^{-1}	0.9	89%
LIR'(Nd/Nd)	862, 870	0.11 % K^{-1}	1.1	98%
LIR''(Nd/Nd)	877, 870	0.25 % K^{-1}	1.2	95%

Section S11 – Cell viability on cancer and healthy cell lines

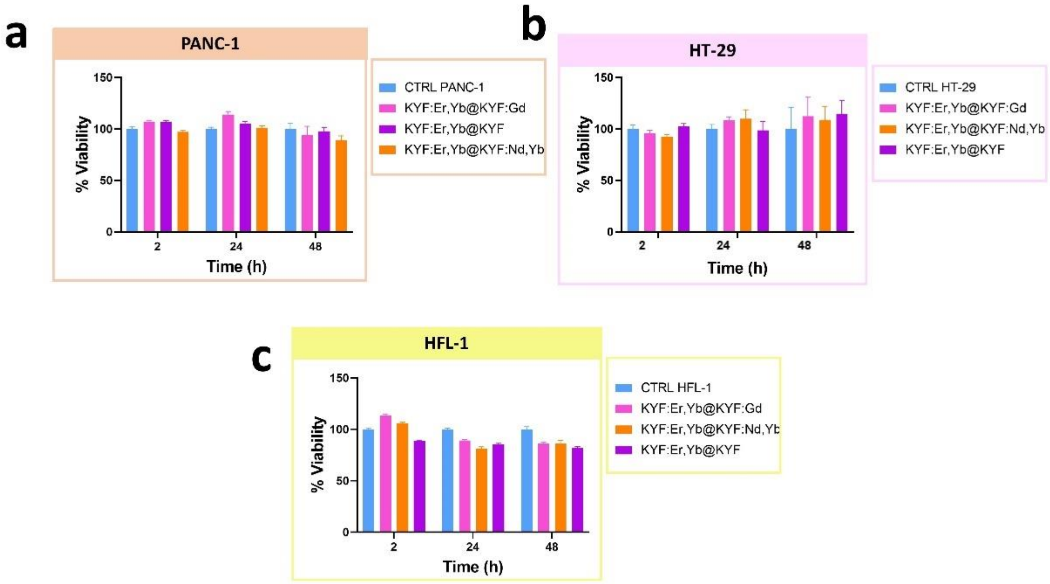

Figure S19. MTT assay for 2 tumoral (PANC-1 and HT-29) and 1 healthy (HLF-1) cell line, at 2, 24 and 48 hours of incubation, for the highest non-toxic concentration.

The MTT assays reveal that KYF:Er,Yb@KYF and KYF:Er,Yb@KYF:Gd are safe up to the concentration of 500 µg/mL in PANC-1, HT-29 and HLF-1 KYF:Er,Yb@KYF:Nd,Yb are safe up to the concentration of 300 µg/mL also after long incubation times (48 h).

Section S12 – X-Rays attenuation evaluated (in HU units) for colloidal dispersion of core@shell nanoparticles in a suitable phantom at different voltages

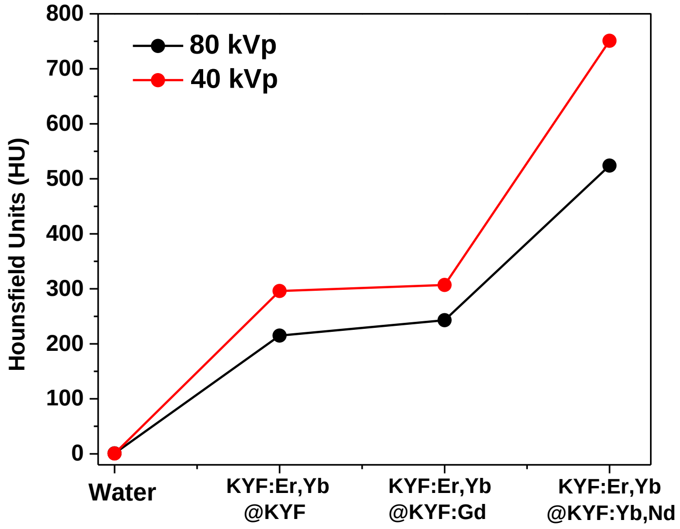

Figure S20. X-Rays attenuation for colloidal dispersions at different nanoparticles concentrations (see main text) at 80 and 40 kVp.

Table T6. CT attenuation values for the three types of core@shell nanoparticles in water dispersion at different concentrations measured at different voltages and some average HU values for most common biological materials, (nd: not defined).

Sample	Concentration	HU@40 kVp	HU@80 kVp	Reference
CS@Y	25 mg/ml	296	215	This work
CS@Gd	35 mg/ml	307	243	This work
CS@Yb,Nd	70 mg/ml	751	524	This work
Bones	nd	1000		(Kim et al., 2017)
Soft tissues	nd	80		(Kim et al., 2017)
Brain	nd	30		(Kim et al., 2017)
Muscles	nd	25		(Kim et al., 2017)
Water	nd	0		(Kim et al., 2017)
Lung	nd	-750		(Kim et al., 2017)
Air	nd	-1000		(Kim et al., 2017)

Bibliography

Hammond, C. (2015). *The Basics of crystallography and Diffraction* (Fourth Edi). Oxford Science Publications.

Kim, J., Chhour, P., Hsu, J., Litt, H. I., Ferrari, V. A., Popovtzer, R., & Cormode, D. P. (2017). Use of Nanoparticle Contrast Agents for Cell Tracking with Computed Tomography. *Bioconjugate Chemistry*, *28*(6), 1581–1597. https://doi.org/10.1021/acs.bioconjchem.7b00194

Li, Y., Natakorn, S., Chen, Y., Safar, M., Cunningham, M., Tian, J., & Li, D. D. U. (2020). Investigations on Average Fluorescence Lifetimes for Visualizing Multi-Exponential Decays. *Frontiers in Physics*, *8*(October). https://doi.org/10.3389/fphy.2020.576862

Pollnau, M., Gamelin, D., Lüthi, S., Güdel, H., & Hehlen, M. (2000). Power dependence of upconversion luminescence in lanthanide and transition-metal-ion systems. *Physical Review B - Condensed Matter and Materials Physics*, *61*(5), 3337–3346. https://doi.org/10.1103/PhysRevB.61.3337

Solanki, P. S., Balabhadra, S., Reid, M. F., Golovko, V. B., & Wells, J. R. (2021). *Upconversion Thermometry Using Yb 3 + / Er 3 + Co-Doped KY 3 F 10 Nanoparticles*.

Vetrone, F., Naccache, R., Zamarrón, A., De La Fuente, A. J., Sanz-Rodríguez, F., Maestro, L. M., Rodriguez, E. M., Jaque, D., Sole, J. G., & Capobianco, J. A. (2010). Temperature sensing using fluorescent nanothermometers. *ACS Nano*, *4*(6), 3254–3258. https://doi.org/10.1021/nn100244a

Würth, C., Kaiser, M., Wilhelm, S., Grauel, B., Hirsch, T., & Resch-Genger, U. (2017). Excitation power dependent population pathways and absolute quantum yields of upconversion nanoparticles in different solvents. *Nanoscale*, *9*(12), 4283–4294. https://doi.org/10.1039/c7nr00092h
